# The effect of bovine dairy products and their components on the incidence and natural history of infection: a systematic literature review

**DOI:** 10.1186/s12937-024-00923-7

**Published:** 2024-02-27

**Authors:** Meghan Mitchell, Mina Suh, Naushin Hooda, Lauren C. Bylsma, Sarah S. Cohen

**Affiliations:** 1 EpidStrategies, a division of ToxStrategies, LLC, 23501 Cinco Ranch Blvd, Suite B226, Katy, TX 77494 USA; 2 Independent contractor to ToxStrategies, LLC, Durham, NC USA

**Keywords:** Dairy, Probiotics, Lactoferrin, Infectious disease, Infection, Incidence, Disease course, Immune response, Leukocytes, Cytokines

## Abstract

**Background:**

Dairy products and their components may impact immune function, although the current evidence base has some research gaps. As part of a larger systematic literature review of dairy products/components (including probiotics, dairy proteins, and dairy fats) and immune function, we identified the available epidemiologic research on the impact of dairy products/components on incidence and natural history of infectious diseases.

**Methods:**

PubMed and Embase databases were systematically searched through May 2022 to identify eligible studies using pre-defined Population, Intervention, Comparator, Outcomes, and Study design criteria. Herein, we focused on describing the impacts of dairy product/component on infectious disease outcomes, including the effect on leukocyte and cytokine response in humans. Risk of bias assessment was performed using the Academy of Nutrition and Dietetics Quality Criteria Checklist. The Preferred Reporting Items for Systematic Reviews and Meta-analyses (PRISMA) guidelines were followed.

**Results:**

Among 9,832 studies identified from the larger literature search, 133 relevant publications from 128 studies reported on dairy product/component and infectious disease outcomes. Few studies are available on the impact of non-fermented milk and traditional yogurt on infectious disease. Evidence was identified to suggest milk and yogurt drinks fermented with *Lactobacillus* strains reduce the risk and burden of common infectious diseases (CIDs), although the findings are mixed and difficult to reconcile due to heterogenous study populations, bacterial strains, and study methods. Few studies are available on the impact of dairy products/components on the natural history of infection, with the available findings indicating probiotics may both improve gastrointestinal symptoms among HIV-infected persons and help eradicate and alleviate the symptoms of *Heliobacter (H.) pylori*. The available evidence also suggests lactoferrin may reduce the virological burden of COVID-19 and hepatitis C virus. No consistent changes in leukocytes or cytokine production were observed for any type of dairy product or their components, but probiotics appeared to enhance natural killer cell levels/activity and the phagocytic process.

**Conclusions:**

Dairy products, particularly those with added probiotics, may represent an easily accessible nutritional intervention to prevent and improve the course of infectious diseases. This review highlights the need for additional research in this potentially impactful area.

**Prospero registration:**

CRD42022333780.

**Supplementary Information:**

The online version contains supplementary material available at 10.1186/s12937-024-00923-7.

## Introduction

Infectious diseases are responsible for substantial morbidity, mortality and economic impacts worldwide (e.g., [1, 2]). A new era of infectious disease transmission dynamics has been described, defined by outbreaks of emerging, re-emerging, and endemic pathogens that are being transmitted rapidly due to global connectivity [3]. Furthermore, an increased incidence of viruses such as influenza and respiratory syncytial virus has been described in the wake of the COVID-19 pandemic, due to the “immunity debt” created by the non-pharmaceutical interventions to control the spread of COVID-19 [4]. As the demographics in the United States shift toward an older population, the impact of these infectious diseases may also be enhanced due to age-related decrements in immune function [5]. Given the substantial burden of infectious diseases and shifting transmission dynamics, it is important to find easy and accessible measures for infection prevention. These measures could be particularly useful to combat “immunity debt” in the wake of another global pandemic and in communal settings of vulnerable populations, such as long-term care facilities for elderly persons and daycare and school settings.

An evolving field of research considers whether the consumption of specific food components has immunostimulatory effects [6, 7]. As an integral part of a healthy dietary pattern [8], dairy products and their components could represent an important and easily modifiable factor to prevent infection in both general and immunocompromised populations. The potential immune-modulating effect of dairy product components has been considered largely in animal and cell models and to a lesser extent in humans [6, 9]. The cumulative findings suggest a beneficial role of dairy products for immune-related outcomes overall; however, studies are heterogeneous in design and quality [10].

While reviews and meta-analyses have examined some dairy products/components in relation to immune-related outcomes, the full scope of this topic is undefined and the findings are described in the literature as insufficient and heterogenous [10–16]. Herein, we sought to identify the full scope of existing literature on this topic and to examine whether dairy products and/or their bioactive components are associated with any aspect of immune function in humans. During our systematic assessment of outcomes that have been examined in the published literature to date, the incidence and natural history of infectious diseases was identified as an outcome with available evidence sufficient to review. Given the increased focus on infectious disease transmission with the COVID-19 pandemic and the substantial morbidity associated with common infectious diseases (CIDs), this SLR examined the available evidence on the potential for dairy products/components to impact the risk of acquiring an infectious disease or affect the natural history of an infectious disease. To complement this assessment, we also evaluated the evidence for the effects of dairy products/components on leukocyte and cytokine response, as these cellular effects are an important component of the pathogenesis of infectious diseases.

The goal of this systematic review is to provide a central location to weigh the epidemiologic evidence on the potential for dairy products/components to impact infectious disease risk/natural history.

## Methods

The study protocol was registered on Prospective Register of Systematic Reviews (PROSPERO, CRD42022333780) (www.crd.york.ac.uk/PROSPERO) prior to the start of this SLR. In the design, execution, and reporting of the current SLR, we followed all Preferred Reporting Items for Systematic Reviews and Meta-analyses (PRISMA) guidelines; the PRISMA checklist is provided as Additional file 1 [17]. The overall SLR was conducted to 1) identify the available evidence on dairy products/components and immune-related outcomes and 2) assess the breadth of the available evidence on the identified outcomes. As the studies resulting from the overall literature review were incredibly heterogeneous in scope, we narrowed the focus of the current manuscript to outcomes related to infectious diseases. Herein, we summarize the evidence related to nutritional interventions with dairy products/components and 1) the incidence of infectious diseases, 2) the natural history of infectious diseases and 3) the impact on leukocyte and cytokine response. Other outcomes related to immune function will be presented in future publications.

### Eligibility criteria

Pre-defined study population, intervention, comparator, outcomes, and study design (PICOS) criteria were used to assess study eligibility.

#### Study population

All studies of humans without dairy sensitivities were included; there were no restrictions on geographical location, sex, age, or health status.

#### Intervention

Exposures/interventions of interest included the following:

1. Cow’s milk products recommended by the United States Department of Agriculture (USDA) in their most recent 2020 guidance, i.e., milk (including milk powders), yogurt, and cheese [18], including those with added traditional and experimental probiotic strains;
2. Cow’s milk proteins (i.e., whey and casein proteins) and peptides;
3. The fat components of milk (i.e., milk phospholipids and the MFGM); and
4. Estimated dairy intake as measured by dietary recall instruments.

Prenatal and maternal exposures to dairy products/components in relation to pediatric outcomes were considered. This review did not evaluate the impact of probiotic strains administered outside the context of dairy products (e.g., as isolated supplements or powders). Studies of bovine colostrum, non-bovine milks, hyperimmunized milk, and raw/unpasteurized milk were excluded. Studies where dairy products/components were administered through a feeding tube were included, but studies using a jejunal tube or other system bypassing the stomach were excluded. Likewise, studies involving the administration of a dairy product/component through a non-oral route (i.e., intranasal, topical, ophthalmic) were excluded. In this review, yogurts using the traditional starter cultures *Lactobacillus (L.) bulgaricus* and *Streptococcus (S.) thermophilus* are referred to as traditional yogurt, while probiotic yogurts are those with additional bacterial strains added.

#### Comparator

Studies with comparison group(s) of low or no dairy product/component consumption or studies comparing pre- and post-intervention outcomes were included. Studies that provided relevant data but did not calculate an effect estimate or conduct any statistical comparisons were excluded.

#### Outcomes

All studies with outcomes related to immune function were included, excluding outcomes related to milk allergies, milk sensitivities, or antibodies to milk proteins. This review summarizes the following outcomes reported in the included studies: 1) the incidence of infectious diseases, 2) the natural history of infectious diseases, 3) leukocyte response and 4) cytokine response. Data on some cytokines, i.e., the small proteins triggered by infection, were excluded from our review since previous SLRs have summarized the impact of dairy products/components on these biomarkers of inflammation, including adiponectin, c-reactive protein (CRP), homocysteine, interleukin (IL)-1, IL-6, IL-8, IL-15, IL-18, intercellular adhesion molecule (ICAM), monocyte chemoattract protein (MCP)-1/CCL2, serum amyloid A (SAA), tumor necrosis factor (TNF), and vascular cellular adhesion molecule (VCAM) [12, 13]. Given the pleiotropic nature of cytokines in general and interleukins specifically (i.e., they could have both inflammatory and non-inflammatory effects), all other cytokines were included. Viral and bacterial disease outcomes were included while fungal infections were excluded.

#### Study design

All observational studies and clinical trials were included. Studies not published in English, conference abstracts, meta-analyses, and case reports or case-series with ≤20 cases were excluded. If more than one article from the same study population was published, data from the publication with the longest follow-up or most relevant population and/or outcomes were evaluated.

### Study identification, screening, and abstraction

The PubMed and Embase databases were used to identify relevant studies published through May 19, 2022, as described in Supplemental Table 1 (Additional file 3). These citations were combined using DistillerSR software [19], which was used to manage the citations during all levels of review and data extraction. One researcher reviewed the titles and abstracts using the PICOS guidance. If an abstract was considered of potential interest, the full-text article was considered by two independent reviewers, with conflicts between the two reviewers resolved by discussion or a senior reviewer. Once a study was identified as relevant at the full-text level, select data were abstracted into DistillerSR. Abstraction elements included study characteristics (e.g., study design, time period of recruitment, and location), population characteristics (e.g., age and health status), information on the intervention(s) and controls (e.g., exposure details, dose and duration), and results related to the relevant outcomes. The abstracted data were assessed by a second reviewer for quality control; conflicts were resolved by a senior reviewer.

**Table 1:** Characteristics of included studies, organized by outcome, dairy exposure, and study quality (*N*=45)

**Author (Year)**	**Study Design**	**Geographical Location**	**Study Period**	**Dairy Product or Component**	**Study Outcome**	**Study Quality**
Shinohara et al. (2020) [20]	Clinical trial	Japan	NR	Milk	Incidence: URTI	Neutral
Turchet et al. (2003) [21]	Clinical trial	Italy	NR	Fermented milk with traditional ferments and *L. casei* DN-114 001	Incidence: winter infections	Negative
Nagata et al. (2011) [22]	Clinical trial	Japan	Both enrollment and follow-up: 2006	Fermented milk with *L. casei* Shirota	Incidence: norovirus gastroenteritis	Positive
Fukushima et al. (2007) [23]	Clinical trial	Japan	Both enrollment and follow-up: 2002-2003	Fermented milk with *L. johnsonii* La1 (NCC533) and *S. thermophilus*	Incidence: infection requiring antibiotics	Positive
Corsello et al. (2017) [24]	Clinical trial	Italy	Both enrollment and follow-up: 2014-2015	Fermented milk with *L. paracasei* CBA L74	Incidence: CID	Positive
Nocerino et al. (2017) [25]	Clinical trial	Italy	Both enrollment and follow-up: 2012	Fermented milk with *L. paracasei* CBA L74	Incidence: CID	Positive
Kinoshita et al. (2019) [26]	Clinical trial	Japan	Enrollment: 2016; Follow-up: 2016-2017	Traditional yogurt	Incidence: common cold or influenza	Positive
Makino et al. (2010) [27]	Clinical trial	Japan	Both enrollment and follow-up: 2005 (Funagata) or 2007 (Arita)	Traditional yogurt	Incidence: common cold or influenza Natural history: *H. pylori*	Positive
Meng et al. (2016) [28]	Clinical trial	United States	Both enrollment and follow-up: 2012-2014	Traditional yogurt and probiotic yogurt with *B. animalis* subsp. *lactis* BB‑12	Incidence: common cold or influenza	Positive
Pu et al. (2017) [29]	Clinical trial	China	Both enrollment and follow-up: 2013	Probiotic yogurt with *L. paracasei* N1115	Incidence: URTI	Positive
Zhang et al. (2021) [30]	Clinical trial	China	NR	*B. animalis* subsp. *lactis* Bl-04	Incidence: Common cold and influenza-like illness	Neutral
Guillemard et al. (2010a) [31]	Clinical trial	Germany	NR	Traditional ferments and *L. casei* DN-114 001	Incidence: CID, including URTI, LRTI and GITI	Positive
Guillemard et al. (2010b) [32]	Clinical trial	Germany	NR	Traditional ferments and *L. casei* DN-114 001	Incidence: CID, including URTI, LRTI, influenza and GITI	Positive
Merenstein et al. (2010) [33]	Clinical trial	United States	NR	Traditional ferments and *L. casei* DN-114 001	Incidence: CID, including GITI, LRTI, and URTI	Positive
Tiollier et al. (2007) [34]	Clinical trial	France	NR	Traditional ferments and *L. casei* DN-114 001	Incidence: RTI	Neutral
Vaisberg et al. (2019) [35]	Clinical trial	Brazil	NR	*L. casei* Shirota	Incidence: URTI	Positive
Van Puyenbroeck (2012) [36]	Clinical trial	Belgium	Both enrollment and follow-up: 2007-2008	*L. casei* Shirota	Incidence: RTI	Neutral
Shida et al. (2017) [37]	Clinical trial	Japan	Both enrollment and follow-up: 2012-2013	*L. casei* Shirota	Incidence: URTI	Positive
Hatakka et al. (2001) [38]	Clinical trial	Finland	NR	*L. rhamnosus* GG	Incidence: RTI	Positive
Sugimura et al. (2015) [39]	Clinical trial	Japan	Both enrollment and follow-up: 2013	*Lactococcus lactis* ssp. *lactis* JCM5805	Incidence: common cold or influenza	Neutral
Zhang et al. (2018) [40]	Clinical trial	China	NR	*L. paracasei, L. casei* 431*, L. fermentium* PCC	Incidence: URTI, influenza-like illness	Neutral
Coman et al. (2017) [41]	Clinical trial	Italy	NR	*L. rhamnosus* IMC 501 and *L. paracasei* IMC 502	Incidence: respiratory symptoms	Negative
Perez et al. (2010) [42]	Clinical trial	Argentina	Both enrollment and follow-up: 2006-2007	*L. casei* CRL431* and L. acidophilus*	Incidence: URTI, gastroenteritis, varicella, and pneumonia	Positive
Kaido et al. (2012 [43])	Retrospective cohort	Japan	Enrollment: 2009-2011	Hydrolyzed whey peptides	Incidence: bacteremia	Neutral
Vitetta et al. (2013) [44]	Clinical trial	Australia	Both enrollment and follow-up: 2008-2010	Bovine lactoferrin/whey protein Ig-rich fraction (Lf/IgF)	Incidence: common cold	Positive
Oda et al. (2021) [45]	Clinical trial	Japan	Both enrollment and follow-up: 2017	Bovine lactoferrin	Incidence: infectious diseases	Neutral
King et al. (2007) [46]	Clinical trial	United States	NR	Bovine lactoferrin	Incidence: URTI, AOM, LRTI	Positive
Kaur and Gathwala (2015) [47]	Clinical trial	India	Enrollment: 2012-2013	Bovine lactoferrin	Incidence: sepsis	Positive
Akin et al. (2014) [48]	Clinical trial	Turkey	Both enrollment and follow-up: 2009-2011	Bovine lactoferrin	Incidence: sepsis	Positive
Manzoni et al. (2009) [49]	Clinical trial	Italy	Enrollment: 2007	Bovine lactoferrin	Incidence: Bacterial and fungal late-onset sepsis	Positive
Darand et al. (2022) [50]	Prospective cohort	Iran	Enrollment: 2014-2016	Estimated intake of dairy, milk, yogurt and cheese from food frequency questionnaire	COVID-19 seroprevalence	Positive
Deschasaux-Tanguy et al. (2021) [51]	Prospective cohort	France	Enrollment: 2009 and on-going Follow-up: 2020	Estimated intake of dairy, milk, yogurt and cheese	COVID-19 seroprevalence	Positive
Cameron et al. (2004) [52]	Case-control	Australia	Both enrollment and follow-up: 2000-2001	Estimated intake of milk and cheese	*Campylobacter jejuni* infection	Positive
Yordanov et al. (2017) [53]	Cross-sectional	Bulgaria	NR	Estimated intake of yogurt	*H. pylori* seroprevalence	Neutral
Ishizaki et al. (2017) [54]	Clinical trial	Vietnam	Both enrollment and follow-up: 2012	Fermented milk with *L. casei* Shirota	Natural history: HIV	Neutral
Irvine et al. (2010) [55]/Irvine et al. (2011) [56]	Retrospective cohort	Tanzania	Both enrollment and follow-up: 2008	Probiotic yogurt with *L. rhamnosus* GR-1 (Fiti)	Natural history: HIV	Neutral
Hummelen et al. (2011) [57]	Clinical trial	Tanzania	Both enrollment and follow-up: 2008	*L. rhamnosus* GR-1	Natural history: HIV	Positive
Yoon et al. (2019) [58]	Clinical trial	South Korea	NR	*L. paracasei* HP7,* Glycyrrhiza glabra*	Natural history: *H. pylori*	Positive
Felley et al. (2001) [59]	Clinical trial	Switzerland	NR	*L. johnsonii* La1	Natural history: *H. pylori*	Positive
Algahtani et al. (2021) [60]	Clinical trial	Egypt	Enrollment: 2020	Bovine lactoferrin	Natural history: COVID-19	Positive
Rosa et al. (2021) [61]	Retrospective cohort	Italy	Enrollment: 2020-2021	Bovine lactoferrin	Natural history: COVID-19	Positive
Campione et al. (2021) [62]	Clinical trial	Italy	Both enrollment and follow-up: 2020	Bovine lactoferrin	Natural history: COVID-19	Neutral
Ueno et al. (2006) [63]	Clinical trial	Japan	Enrollment: 2001, Interim analysis: 2004	Bovine lactoferrin	Natural history: HCV	Positive
Kaito et al. (2007) [64]	Clinical trial	Japan	Enrollment: 2009-2011	Bovine lactoferrin	Natural history: HCV	Positive
Ishibashi et al. (2005) [65]	Clinical trial	Japan	Both enrollment and follow-up: 2002-2004	Bovine lactoferrin	Natural history: HCV	Positive

*AOM* Acute otitis media, *CID* Common infectious disease, *GITI* Gastrointestinal tract infection, *HCV* Hepatitis C virus *LRTI* Lower respiratory tract infection, *RTI* Respiratory tract infection, *URTI* Upper respiratory tract infection

Tables 2, 3, 4, 5 and 6 and Supplemental Tables 3 and 4 summarize the effect measures and/or relevant statistical comparisons in the identified studies. If a comparison was not statistically significant, we indicated what specific outcome was measured, which groups were compared and that the difference was not statistically significant (NSS). If a comparison was statistically significant, we indicated what specific outcome was measured, which groups were compared, the direction of the change, and that the difference was statistically significant (SS) with a *p*-value. Hazard ratios (HRs), relative risks (RRs) and odds ratios (ORs) were reported with the 95 percent confidence interval (95% CI), where appropriate. In Supplemental Tables 3 and 4 (Additional file 3), hierarchies were created to be parsimonious in reporting the most meaningful results: comparisons between the experimental and control groups were chosen over comparisons within these groups; comparisons with the highest vs. the lowest dose were chosen (i.e., tertile 3 vs. tertile 1); and, the most adjusted comparison was chosen if multiple models were available.

**Table 2:** Clinical trials of dairy products/probiotics on the incidence, duration, and severity of acute infections (*N*=23)

**Author (Year)**	**Exposure(s) being studied**	**Study Population**		**Dairy intervention details**	**Infectious disease**	**Measures of infection risk**	**Duration and/or severity of infection or symptoms**
		**N**	**Age, gender and health status**				
**Exposure - whole dairy products (by product-milk, fermented milk, traditional yogurt, and probiotic yogurt)**							
Shinohara et al. (2020) [20]	Milk	Exp: 8 Control: 5	Adults, healthy	Exp: 250 mL of milk once a week during bowling exercise for one year Control: 250 mL sports drink during bowling exercise once a week for one year	URTI based on questionnaire	Average incident cases: SS decrease **(*p*<0.01)** exp vs. control SS inverse correlation **(*p*=0.03)** between total dairy consumption and URTI incidence	SS inverse correlation **(*p*=0.01)** between total dairy consumption and URTI severity
Turchet et al. (2003) [21]	Fermented milk with traditional ferments and *L. casei* DN-114 001 (Actimel®)	Exp: 180 Control: 180	Adults, healthy	Exp: 100 mL Actimel®, fermented milk with traditional ferments and *L. casei* DN-114 001, twice daily for 3 weeks Control: none	Clinically verified winter infections	Cumulative incidence: Differences NSS for all pathologies (*p*=0.662), ENT pathology (*p*=0.248), influenza syndrome (*p*=0.815), gastrointestinal syndrome (*p*=0.836), and bacterial broncho-pneumopathy (*p*=0.240)	Duration of all pathologies: SS decrease **(*p*=0.024)** exp vs. control
Nagata et al. (2011) [22]	Fermented milk with *L. casei* Shirota	Exp: 39 Control: 38	Elderly, hospitalized patients	Exp: 80 mL fermented milk with *L. casei* Shirota once daily until discharge Control: none	Clinically verified norovirus gastroenteritis	Cumulative incidence occurring in winter season: Differences NSS (no *p*-value reported) exp vs. control	Duration of vomiting/diarrhea: Differences NSS (no *p*-value reported) exp vs. control Duration of fever (>37˚C): SS decrease **(*p*=0.027)** exp vs. control Duration of fever (>38˚C): Differences NSS (*p*=0.088) exp vs. control
Fukushima et al. (2007) [23]	Fermented milk with *L. johnsonii* La1 (NCC533) and *S. thermophilus*	Exp: 12 Control: 12	Elderly, hospitalized	Exp: 90 g fermented milk (373 kJ) with *L. johnsonii* La1 (NCC533) and *S. thermophilus* through a tube after feeding of EN (3395 kJ) daily for 12 weeks Control: EN diet at 3395 kJ, then administered 373 kJ of the EN in the same manner as the fermented milk daily for 12 weeks Run-in observation period 12 weeks before starting intervention	Clinically verified infection requiring antibiotic	NR	Mean duration of infection (% of days in 12 weeks): SS difference observation period - intervention **(*p*=0.047)** exp. vs. control (favors treatment) Mean duration of fever (% of days in 12 weeks): Difference observation period - intervention NSS (*p*=0.078) exp. vs. control
Corsello et al. (2017) [24]	Fermented milk with *L. paracasei* CBA L74	Exp: 73 Control: 73	Children attending daycare or preschool, healthy	Exp: 150 mL fermented milk with *L. paracasei* CBA L74 daily for 3 months Control: 150 mL maltodextrins with an energy content similar to that of the fermented milk daily for 3 months	Clinically verified CID, including GITI or URTI	Cumulative proportion with at least one CID: SS decrease **(*p*=0.002)** exp vs. control Proportion of patients with at least one episode of acute gastroenteritis, pharyngitis, laryngitis, tracheitis: SS decrease (**0.007, 0.007, 0.029, 0.048**, respectively) exp. vs. control Negative binomial regression PPA analysis for CID incidence: **IRR=0.64 (95% CI=0.42-0.98)** exp. vs. control	Proportion of patients with at least one medication course: SS decrease **(*p*=0.019)** exp. vs. control Negative binomial regression PPA analysis for lost days of school: **IRR=0.26 (95% CI=0.13-0.53)** exp. vs. control
Nocerino et al. (2017) [25]	Fermented milk with *L. paracasei* CBA L74	Exp: 141 Control: 127	Children attending preschool or daycare, healthy	Exp: 150 mL fermented milk with *L. paracasei* CBA L74 daily for three months Control: 150 mL maltodextrins with an energy content similar to that of the fermented milk daily for 3 months	Clinically verified CID	Proportion with at least one episode of CID: SS decrease (***p*<0.0001**) exp. vs. control Proportion of patients with at least one episode of acute gastroenteritis, rhinitis, otitis, pharyngitis, laryngitis, tracheitis: SS decrease (**<0.0001, 0.003, <0.0001, <0.001, 0.005, 0.018**, respectively) exp. vs. control Position regression PPA for CID incidence: **IRR=0.36 (95% CI=0.29-0.44)** exp. vs. control Binary logistic regression analysis for CID incidence: **OR=0.19 (95% CI=0.11-0.37)** exp. vs. control	Odds of at least one medication course: **OR=0.26 (95% CI=0.15-0.43)** exp. vs. control
Kinoshita et al. (2019) [26]	Traditional yogurt (Meiji Probio Yogurt R1®)	Exp: 479 Control: 482	Female adults, healthy, healthcare workers	Exp: 112 mL of Meiji Probio Yogurt R-1® (*L. delbrueckii* ssp*. bulgaricus* [OLL1073R-1] and *S. thermophilus*) daily for 16 weeks Control: no yogurt	Common cold or influenza based on self-report of physician diagnosis	Cumulative incidence: Differences NSS for influenza (*p*=0.91) and common cold (*p*=0.49) Differences NSS for Kaplan-Meir analysis of influenza and common cold incidence	NR
Makino et al. (2010) [27]	Traditional yogurt (Meiji Probio Yogurt R1)	Exp 1 (Fungata study): 29 Exp 2 (Arita study): 44 Control 1 (Fungata study): 28 Control 2 (Arita study): 43	Elderly, healthy	Exp 1: 90 g of Meiji Probio Yogurt R-1® (*L. delbrueckii* ssp*. bulgaricus* [OLL1073R-1] and *S. thermophilus*) daily for 8 weeks Exp 2: 90 g of Meiji Probio Yogurt R-1 (*L. delbrueckii* ssp*. bulgaricus* [OLL1073R-1] and *S. thermophilus*) daily for 12 weeks Control 1: 100 mL milk daily for 8 weeks Control 2: 100 mL milk daily for 12 weeks	Common cold based on based on questionnaire reviewed by clinician and influenza based on receipt of hospital treatment	Odds of cold or influenza: Fungata: OR = 0.29, *p*=0.103 Arita: OR = 0.44, *p*=0.084 Meta-analysis: **OR = 0.39, *p*=0.019**	NR
Meng et al. (2016) [28]	Traditional yogurt Probiotic yogurt with *B. animalis* subsp*. lactis* BB‑12	30 (cross-over trial)	Adults, healthy	Exp 1: One 8-oz (240 g) serving of yogurt smoothie (BB-12 added pre fermentation) daily for four weeks Exp 2: One 8-oz (240 g) serving of yogurt smoothie (BB-12 added post fermentation) daily for four weeks Exp 3: 1 capsule containing BB-12 daily for four weeks Control: One 8-oz (240 g) serving of yogurt smoothie (no probiotic) daily for four weeks A two-week washout period between treatment periods applied	Common cold or influenza based on questionnaire	Cumulative incident cases of cold or flu: Differences NSS (*p*=0.1709) baseline (1 month before treatments) vs. all treatments in logistic regression model Mean number of cold or flu episodes: Differences NSS (*p*=0.2316) baseline (1 month before treatments) vs. all treatments in Poisson regression model	Duration of URTI symptoms (days): SS decrease baseline vs. exp 1 **(*p*<0.01)** and baseline vs. control **(*p*<0.05)** Number of days in bed or away from work: Differences NSS (*p*=0.42) baseline vs. exp 1, exp 2, or control Sick score due to cold or flu: Differences NSS (*p*=0.06) baseline vs. exp 1, exp 2, or control
Pu et al. (2017) [29]	Probiotic yogurt with *L. paracasei* N1115	Exp: 103 Control: 102	Adults, healthy	Exp: 100 mL of probiotic yogurt three times a day for 12 weeks Control: none	URTI based on questionnaire	Number of URTI events: SS decrease **(*p*=0.030)** exp. vs. control Number of persons with URTI: SS decrease **(*p*=0.038)** exp. vs. control Mean number of URTI episodes per person: SS decrease **(*p*=0.043)** exp. vs. control RR of URTI: RR=**0.55 (95% CI: 0.307–0.969)** exp. vs. control	URTI score: Difference NSS (*p*=0.913) exp. vs. control
**Exposure - probiotics (by genus, species and strain)**							
Zhang et al. (2021) [30]	*B. animalis* subsp. *lactis* Bl-04 [given in yogurt]	Exp: 62 Control: 61	Adults, healthy	Exp: 250 g of Qingrun® yogurt (yogurt drink with *B. animalis* subsp. *lactis* Bl-04, *L casei*, *L. bulgaricus*, and *S. thermophilus*) once daily for 12 weeks Control: 250 g of control yogurt (yogurt drink with *L. casei, L. bulgaricus,* and *S. thermophilus*) once daily for 12 weeks	Common cold and influenza-like illness (URTI) based on questionnaire	OR for common cold: OR=**0.38 (95% CI=0.17-0.81)** OR for influenza-like illness: OR=**0.38 (95% CI=0.17-0.81)** SS difference **(*p*=0.0002)** in frequency distribution of number of URTI episodes	Duration of URTI symptoms (days): SS decrease **(*p* <0.0001)** exp vs. control Severity score of URTI symptoms: SS decreas**e (*p* <0.0001)** exp vs. control Duration of medication due to URTI (days): SS decrease **(*p* <0.0001)** exp vs. control Duration of sick leave due to URTI (days): Difference NSS (*p*=0.433) exp vs. control
Guillemard et al. (2010a) [31]	Traditional ferments and *L. casei* DN-114 001 [given in yogurt drink Actimel®]	Exp: 500 Control: 500	Adults, healthy, shift workers	Exp: 100mL Actimel®, fermented milk (*L. delbrueckii* ssp*. bulgaricus* and *S. thermophilus*) with added *L. casei* DN-114 001, twice daily for 3 months Control: Non-fermented dairy drink at same dose and duration	Clinically verified CID, including URTI, LRTI and GITI	Cumulated number of all CIDs by Poisson regression: RR=0.92 (95% CI=0.78-1.09) exp vs. control Cumulated number of CIDs by logistic regression: OR=**0.75 (95% CI=0.59-0.95)** Proportion with ≥1 CID: SS decrease **(*p*=0.005)** exp vs. control Occurrence of CID by logistic regression: OR=**0.695 (95% CI=0.540-0.896)**	Mean duration of CID episode (days): Difference NSS (*p*=0.182) exp. vs. control Cumulative time with CIDs per subject (days): Difference NSS (*p*=0.084) exp. vs. control Cumulative duration of fever (days):**.022)** exp. vs. control % with severe symptoms: Differences NSS (*p*-value not reported) exp. vs. control CID-associated total medication: Differences NSS (*p*-value not reported) exp. vs. control Duration and occurrence of sick leave due to CID: Differences NSS (*p*-value not reported) exp. vs. control
Guillemard et al. (2010b) [32]	Traditional ferments and *L. casei* DN-114 001 [given in yogurt drink Actimel®]	Exp: 537 Control: 535	Elderly, healthy	Exp: 100mL Actimel®, fermented milk (*L. delbrueckii* ssp*. bulgaricus* and *S. thermophilus*) with added *L. casei* DN-114 001, twice daily for 3 months Control: Non-fermented dairy drink at same dose and duration	Clinically verified CID, including URTI, LRTI, influenza and GITI	Cumulated number of all CIDs by Poisson regression: Differences NSS (*p*-value not reported) Mean CID rate by Poisson regression: RR=0.89 (95% CI=0.70-1.14) exp vs. control	Mean duration per episode: SS decrease exp. vs. control for all CID **(*p*=0.008),** URTI **(*p*=0.0002),** and rhinopharyngitis **(*p*=0.0003)** Cumulative duration: SS decrease exp. vs. control for all CID **(*p*=0.009),** URTI **(*p*=0.0003),** and rhinopharyngitis **(*p*=0.0006)** Severity (use of CID-associated medication) and intensity/duration of fever: Differences NSS (no *p*-value reported) exp. vs. control for each analysis
Merenstein et al. (2010) [33]	Traditional ferments and *L. casei* DN-114 001 [given in yogurt drink DanActive®]	Exp: 314 Control: 324	Children, healthy	Exp: 200 mL strawberry flavored DanActive®, fermented milk (*L. bulgaricus* and *S. thermophilus*) with added *L. casei* DN-114 001, daily for 90 days Control: 200 mL non-fermented dairy drink at same dose and duration	CID, (including GITI, LRTI, URTI) based on parental report	Incidence rate of CIDs per 100-person day: IRR=**0.81 (95% CI=0.65-0.99)** Incidence rate of GITI per 100-person day: IRR=**0.76 (95% CI=0.58-0.99)** Incidence rate of URTI per 100-person day: IRR=**0.82 (95% CI=0.68-0.99)** Incidence rate of LRTI per 100-person day: IRR=0.98 (95% CI=0.82-1.18)	Rate of days with change in activity because of illness per 100-person days: Differences NSS (*p*=0.91) exp. vs. control Rate of vomiting, stomach pain, constipation, runny nose, cough, decreasing appetite, fever and rash per 100-person days: Differences NSS for each analysis (*p*=0.10, 0.36, 0.68, 0.39, 0.36, 0.54, 0.99, 0.21, respectively)
Tiollier et al. (2007) [34]	Traditional ferments and *L. casei* DN-114 001 [given in yogurt drink Actimel®]	Exp: 24 Control: 23	Adult male cadets, healthy	Exp: 100 mL Actimel®, fermented milk (*L. delbrueckii* ssp*. bulgaricus* and *S. thermophilus*) with added *L. casei* DN-114 001, three times daily for 1 month during commando training Control: 100 mL of non-fermented milk three times daily for 1 month during commando training	Clinically verified RTIs	Cumulative number of persons with RTI: Differences NSS (*p*=0.46) exp. vs. control Incidence of RTI: Difference NSS (*p*=0.98) exp. vs. control	Mean number of days with symptoms: Difference NSS (*p*=0.67) exp. vs. control Mean number of symptoms and daily mean number of symptoms: Difference NSS (*p*=0.23, *p*-value=not reported) exp. vs. control Proportion of rhinopharyngitis: SS higher (***p*<0.05**) exp vs. control
Vaisberg et al. (2019) [35]	*L. casei* Shirota [given in Yakult®]	Exp: 20 Control: 22	Adult male marathon runners, healthy	Exp: 80 g of Yakult®, fermented milk with *L. casei* Shirota, daily for 30 days prior to marathon Control: 80 g non-fermented milk daily for 30 days prior to marathon	Upper respiratory symptoms based on self-report	Cumulative proportion with upper respiratory symptoms post-marathon: Differences NSS (*p*=0.076) exp. vs. control	Duration of upper respiratory symptoms post-marathon: Differences NSS (*p*=0.089) exp. vs. control
Van Puyenbroeck (2012) [36]	*L. casei* Shirota [given in milk]	Exp: 375 Control: 362	Elderly institutionalized, healthy	Exp: 65 mL fermented milk with *L. casei* Shirota twice daily for 176 days Control: 65 mL non-fermented milk twice daily for 176 days	Clinically verified RTI	Number of participants with at least one day of symptoms: Difference NSS (*p*=0.325) exp. vs. control Generalized linear mixed modeling with the outcome of one or more respiratory symptoms: OR=0.8715 (95% CI=0.6168- 1.2887) exp vs. control Multivariate logistic regression analysis with the outcome development of a severe RTI: OR=0.592 (95% CI=0.335-1.049)	Number of days of respiratory symptoms: Difference NSS (*p*=0.342) exp. vs. control
Shida et al. (2017) [37]	*L. casei* Shirota [given in Yakult®]	Exp: 49 Control: 47	Adults, healthy	Exp: One bottle of Yakult®, fermented milk with *L. casei* Shirota, daily for 12 weeks Control: One bottle non-fermented milk daily for 12 weeks	Clinically verified URTI, including common cold and influenza	Cumulative proportion of patients with incident URTI: SS decrease **(*p*=0.002)** exp vs. control Cumulative proportion of patients with incident cold: SS decrease **(*p*=0.005)** exp vs. control Cumulative proportion of patients with incident influenza: SS decrease (*p*=0.201) exp vs. control Kaplan Meir time-to-event analysis: SS higher **(*p*=0.0008)** URTI-free rate exp. vs. control Mean cumulative number of URTI episodes: SS **(*p*=0.004)** decrease exp. vs. control	Mean duration of each URTI episode (days): SS decrease **(*p*=0.002)** exp. vs. control Mean cumulative days with URTI symptoms: SS decrease **(*p*=0.001)** exp. vs. control Mean severity score of URTIs: Differences NSS (=0.966) exp vs. control
Hatakka et al. (2001) [38]	*L. rhamnosus* GG [given in milk]	Exp: 282 Control: 289	Children attending daycare, healthy	Exp: Fermented milk with *L. rhamnosus* GG (ATCC 53103) three times daily, five days a week, for 7 months (average consumption 260 mL) Control: Non-fermented milk three times daily, five days a week, for 7 months (average consumption 260 mL)	Clinically verified RTI	Age-adjusted logistic regression all infections, acute otitis media, sinusitis, acute bronchitis, and pneumonia: OR=0.75 (95% CI=0.52-1.09), OR=0.78 (95% CI=0.53-1.14), OR=0.86 (95% CI=0.33-2.22), OR=0.80 (95% CI=0.39-1.64), OR=0.83 (95% CI=0.18-3.78) exp vs. control	Mean ITT age-adjusted duration of total, respiratory, and gastrointestinal symptoms (days): Differences NSS (*p*=0.59, 0.67, 0.74, respectively) exp. vs. control Mean ITT age-adjusted absence due to illness (days): Differences NSS (*p*=0.09) exp. vs. control Mean ITT age-adjusted total symptoms score: Differences NSS (*p*=0.36) exp. vs. control Age-adjusted logistic regression for all antibiotic treatment: OR=0.78 (95% CI=0.54-1.11) Correlation between amount of milk consumed and the total number of days of illness: *r* = − 0.12, *p*=0.07 Correlation between amount of milk consumed and days with respiratory symptoms: r = − 0.11; *p*=0.09 Correlation between amount of milk consumed and days with gastrointestinal symptoms: **r = − 0.17; *p*=0.007**
Sugimura et al. (2015) [39]	*Lactococcus lactis* ssp. *lactis* JCM5805 [given in yogurt drink]	Exp: 106 Control: 107	Adults, healthy	Exp: 100 mL fermented yogurt drink with *Lactococcus lactis* ssp. *lactis* JCM5805 daily for 10 weeks Control: 100 mL non-fermented yogurt drink daily for 10 weeks	Clinically verified common cold or influenza	Cumulative incident influenza or common cold cases: Differences NSS (*p*=0.127) exp. vs. control	Number of days with cough and feverishness: SS decrease **(*p*<0.001 for each)** exp. vs. control Number of days with sore throat and headache: Differences NSS (*p*=0.226 and *p*=0.958, respectively) exp. vs. control Number of days with moderate/severe cough, sore throat and feverishness: SS decrease **(*p*=0.015, *p*=0.009, *p*=0.009,** respectively) exp. vs. control Number of days with moderate/severe headache: Differences NSS (*p*=0.679) exp. vs. control
Zhang et al. (2018) [40]	*L. paracasei, L. casei* 431*, L. fermentium* PCC [in yogurt drink]	Exp: 67 Control: 67	Adults, unclear (history of cold ≥4 times in the past year)	Exp: 150 mL of fermented yogurt drink with *L. paracasei, L. casei* 431*, L. fermentium* PCC once daily for 12 weeks Control: 150 mL of yogurt fermented by starter culture only once daily for 12 weeks	URTI and flu-like illness (no information on method of outcome assessment)	Cumulative proportion with URTI: SS decrease **(*p*=0.002)** exp. vs. control Cumulative proportion flu-like illness with fever: SS decrease **(*p*=0.034)** exp. vs. control Cumulative proportion URTI symptom without fever: SS decrease **(*p*=0.023)** exp. vs. control	Mean duration URTI symptoms (days): SS decrease **(*p*<0.001)** exp vs. control Cumulative proportion receiving drug treatment for URTI symptoms: SS decrease **(*p*<0.001)** exp. vs. control Cumulative proportion missing work: Differences NSS (no *p*-value reported) exp. vs. control Severity scores of URTI symptoms: SS decrease **(*p*=0.028)** exp. vs. control Mean days of medication: Differences NSS (*p*=0.064) exp. vs. control Mean number of sick days: Differences NSS (*p*=0.290) exp. vs. control
Coman et al. (2017) [41]	*L. rhamnosus* IMC 501 *and L. paracasei* IMC 502 [given in milk]	Exp: 5 Control: 5	Adults, healthy	Exp: 200 mL of fermented milk with *L. rhamnosus* IMC 501 and *L. paracasei* IMC 502 once daily for 4 weeks Control: 200 mL of fermented milk with no additional probiotics once daily for 4 weeks	Respiratory symptoms based on questionnaire	Mean change in respiratory symptom scores on Wisconsin Upper Respiratory Symptom Survey: Differences NSS (no *p*-values reported) exp. vs. control for runny nose, nose closed, sneezing, sore throat, irritated throat, cough, hoarseness, head congestion, chest congestion and tiredness	NR
Perez et al. (2010) [42]	*L. casei* CRL431and *L. acidophilus* CRL730 [given in milk]	Exp: 70 Control: 70	Children, healthy, low SES	Exp: 90 g fermented milk with *S. thermophilus*, *L. casei* CRL431and *L. acidophilus* CRL730 once daily for at least 4 months Control: 90 g fermented milk with *S. thermophilus* once daily for at least 4 months	Clinically verified URTI, gastroenteritis, varicella, and pneumonia	Number of patients with URTI, gastroenteritis, varicella, and pneumonia: Differences NSS (0.882, 0.326, 0.476 and 1.00, respectively) exp. vs. control	Days of fever: Differences NSS (*p*=0.235) exp. vs. control

*AOM *acute otitis media, *CID *common infectious disease, *EN *enteral nutrition, *ENT *ear, nose and throat, *GITI *gastrointestinal tract infection, *IRR *incidence rate ratio, *ITT *intent-to-treat, *LRTI *lower respiratory tract infection, *NR *not reported, *NSS *not statistically significant, *OR* odds ratio, *PPA *per protocol analysis, *RTI *respiratory tract infection, *SS *statistically significant, *URTI*, upper respiratory tract infection

Statistical comparisons that were significant at the *p*=0.05 level are bolded

**Table 3:** Studies of dairy proteins on incidence, duration, and severity of acute infections (*N*=7)

**Author (Year)**	**Exposure(s) being studied**	**Study population**		**Dairy intervention details**	**Infectious disease**	**Measures of infection risk**	**Duration and/or severity of infection or symptoms**
		**N**	**Age, gender and health status**				
Kaido et al. (2012) [43]	Hydrolyzed whey peptide	Exp: 40 Control: 36	Adults, post-liver transplant	Exp: Immune-modulating diet enriched with hydrolyzed whey peptide started within the first 24 h after surgery through a jejunostomy tube infused at 20-40 ml/h for 10-14 days Control: Conventional diet started within the first 24 h after surgery through a jejunostomy tube infused at 20-40 ml/h for 10-14 days	Clinically verified bacteremia	Proportion with bacteremia: SS decrease **(*p*=0.002)** exp. vs. control In-hospital death due to infection: Differences NSS (*p*=0.145) exp. vs. control	NR
Vitetta et al. (2013) [44]	Bovine lactoferrin/whey protein Ig-rich fraction	Exp: 53 Control: 52	Adults, at least 3 cold events in the past 6 months	Exp: Two 300 mg capsules (containing 200 mg of lactoferrin and 100 mg of IgF) daily for 90 days Control: Two 300 mg capsules (calcium phosphate) daily for 90 days	Common cold based on self-report of symptoms	Mean cold events 1-90 days: SS decrease (***p* <0.001)** exp. vs. control Mean cold events 1-45 days: SS decrease **(*p* <0.001)** exp. vs. control Mean cold events 46-90 days: SS decrease **(*p* <0.001)** exp. vs. control Total number of symptoms associated with a cold 1-90 days: SS decrease **(*p* < 0.05)** exp. vs. control	Median days ill at first follow-up (day 45): Difference NSS (*p*=0.10) exp. vs. control Median days ill at second follow-up (day 90): Difference NSS (*p*=0.49) exp. vs. control Median cold event severity at first follow-up (day 45): Difference NSS (*p*=0.76) exp. vs. control Median cold event severity at second follow-up (day 90): Difference NSS (*p*=0.08) exp. vs. control
King et al. (2007) [46]	Bovine lactoferrin	Exp: 26 Control: 26	Infants ≤4 weeks of age, healthy	Exp: Similac iron formula with 850 mg/L bovine lactoferrin for 12 months Control: Regular cow milk based Similac iron formula (102 mg/L bovine lactoferrin) for 12 months	Clinically confirmed URTI, AOM, LRTI	Mean episodes/infant-year: Differences NSS (p-vales not reported) exp. vs. control for URTI, AOM, and other illnesses Mean episodes/infant-year, LRTI: SS decrease (***p*<0.05**) exp. vs. control	Mean duration (days): Differences NSS (*p*-values not reported) for URTI, AOM, LRTI, and other illnesses
Kaur and Gathwala (2015) [47]	Bovine lactoferrin	Exp: 63 Control: 67	Infants, low birth weight and hospitalized	Exp: 100-250 mg bovine lactoferrin (based on weight) dissolved in milk daily from 1st to 28th day of life Control: Placebo (Glucon D) dissolved in milk daily from 1st to 28th day of life	Clinically confirmed late-onset sepsis	RR of culture-proven sepsis: RR=0.211(95% CI=0.044–1.019) exp. vs. control **(*p*=0.036)** RR of bacterial sepsis: RR=0.242 (95% CI=0.049–1.186) (*p*= 0.061) RR of probable sepsis: RR=**0.257 (95% CI=0.08-0.828)** exp. vs. control **(*p*=0.016)** RR of any sepsis: RR=**0.201 (95% CI=0.076-0.537)** exp. vs. control **(*p*=0.001)** Sepsis-attributable mortality: SS decrease (***p*=0.027**) exp. vs. control	NR
Akin et al. (2014) [48]	Bovine lactoferrin	Exp 1: 25 Control 1: 22	Infants, preterm and/or very low birth weight and hospitalized	Exp 1: 200 mg lactoferrin daily, after the baby reached 20 mL/kg/d feeding volume and continued throughout the hospitalization period Control 1: 2 ml saline once a day, after the baby reached 20 mL/kg/d feeding volume and continued throughout the hospitalization period	Clinically confirmed sepsis	Number of patients with sepsis: Difference NSS (*p*=0.572) exp. vs. control Number of sepsis attacks per 1,000 patient days: SS decrease (***p*=0.007**) exp. vs. control	NR
Manzoni et al. (2009) [49]	Bovine lactoferrin and *Lactobacillus rhamnosus* GG	Exp 1: 153 Exp 2: 151 Control: 168	Infants, very low birth weight and hospitalized	Exp 1: 100 mg lactoferrin daily from birth to 30^th^ day of life Exp 2: 100 mg lactoferrin and *Lactobacillus rhamnosus* GG daily from birth to 30^th^ day of life Control: Placebo daily from birth to 30^th^ day of life	Clinically confirmed late-onset sepsis	RR of late-onset sepsis: **RR=0.34 (95% CI=0.17-0.70)** exp 1 vs. control RR of late-onset sepsis: **RR=0.27 (95% CI=0.12-0.60)** exp 2 vs. control Mortality attributable to sepsis: exp 1 vs. control, *p*=0.008 Mortality attributable to sepsis: RR=0.14 (0.02-1.09) exp 2 vs. control, ***p*=0.04** Multivariable logistic regression: **OR=0.32 (95% CI=0.14-0.77)** exp 1 vs. control **OR=0.21 (0.08-0.55)** exp 2 vs. control	NR
Oda et al. (2021) [45]	Bovine lactoferrin	Exp 1: 103 Exp 2: 103 Control: 104	Adults, healthy	Exp 1: 200 mg lactoferrin daily for 12 weeks Exp 2: 600 mg lactoferrin daily for 12 weeks Control: Placebo tablets daily for 12 weeks	Clinically confirmed infectious diseases, including summer colds, gastroenteritis, colds sores and styes	Prevalence of infectious diseases: Difference NSS (*p*=0.203) exp 1 vs. control Difference NSS (*p*=0.240) exp 2 vs. control p-trend= 0.240 Prevalence of summer colds: Difference NSS (*p*=0.488) exp 1 vs. control Difference NSS (*p*=0.571) exp 2 vs. control p-trend= 0.571 Median number of episodes, total infectious diseases: Difference NSS (*p*=0.348) exp 1 vs. control Difference NSS (*p*=0.673) exp 2 vs. control p-trend= 0.612 Median number of episodes, summer colds: Difference NSS (*p*=0.857) exp 1 vs. control Difference NSS (*p*=0.804) exp 2 vs. control p-trend= 0.832 Prevalence of summer cold symptoms: Differences NSS (p=0.170, 0.243, 0.895, 0.401, 0.685, 0.305 and 0.571, respectively) for sore throat, cough, nasal secretion, nasal congestion, headache, chills, and fatigue	Median duration, total infectious diseases: SS decrease **(*p*=0.045)** exp 1 vs. control SS decrease **(*p*=0.010)** exp 2 vs. control p-trend=**0.011** Median duration, summer cold: Difference NSS (*p*=0.204) exp 1 vs. control SS decrease **(*p*=0.036)** exp 2 vs. control p-trend= 0.060 Median duration of cold sores, gastroenteritis, styes: Differences NSS (*p*-values not reported) exp 1 and 2 vs. control Median duration of summer cold symptoms: Differences NSS (*p*=0.096, 0.196, 0.283, 0.884, 0.657, 0.599, and 0.095, respectively) for sore throat, cough, nasal secretion, nasal congestion, headache, chills, and fatigue Number of medications, total infectious diseases: Difference NSS (*p*=0.561) exp 1 vs. control Difference NSS (*p*=0.910) exp 2 vs. control p-trend=0.873 Number of medications, summer colds: Difference NSS (*p*=0.736) exp 1 vs. control Difference NSS (*p*=0.895) exp 2 vs. control p-trend=0.913 Median duration of medication, total infectious diseases: Difference NSS (*p*=0.352) exp 1 vs. control Difference NSS (*p*=0.120) exp 2 vs. control p-trend=0.085 Median duration of medication, summer colds: Difference NSS (*p*=0.460) exp 1 vs. control Difference NSS (*p*=0.082) exp 2 vs. control p-trend=0.053

*AOM *acute otitis media, *CID *common infectious disease, *EN *enteral nutrition, *ENT *ear, nose and throat, *GITI *gastrointestinal tract infection, *IRR *incidence rate ratio, *ITT *intent-to-treat, *LRTI *lower respiratory tract infection, *NSS *not statistically significant, *OR *odds ratio, *PPA *per protocol analysis, *RTI *respiratory tract infection, *SS *statistically significant, *URTI *upper respiratory tract infection

Statistical comparisons that were significant at the *p*=0.05 level are bolded

**Table 4:** Literature on the effects of dietary patterns involving dairy on acute infections (*N*=4)

**Author (Year)**	**Study population**		**Exposure details**	**Infectious disease**	**Measures of infection risk**
	**N**	**Age, gender, and health status**			
Darand et al. (2022) [50]	8,801	Adults, healthy	Food frequency questionnaire evaluated intakes in the previous year of the following: Total dairy (total, low-fat and high-fat) Milk (total, low-fat and high-fat) Yogurt (total, low-fat and high-fat) Cheese	COVID-19 seroprevalence	Multivariate logistic regression: Low-fat dairy: Tertile 3=OR=**0.51 (95% CI=0.37-0.69)** p-trend (tertiles 2 and 3)=**<0.001** High-fat dairy: Tertile 3=OR=**1.40 (95% CI=1.09-1.92)** p-trend=**0.03** Total dairy: Tertile 3=OR=1.03 (95% CI=0.76-1.39) p-trend=0.97 Low-fat milk: Tertile 3=OR=**0.47 (95% CI=0.35-0.59)** p-trend=**<0.001** High-fat milk: Tertile 3=OR=**1.54 (95% CI=1.20-1.97)** p-trend=<0.001 Total milk: Tertile 3=OR=0.74 (95% CI=0.54-1.01) p-trend=0.06 Low-fat yogurt: Tertile 3=OR=1.12 (95% CI=0.82-1.52) p-trend=0.31 High-fat yogurt: Tertile 3=OR=1.21 (95% CI=0.93-1.59) p-trend=0.27 Total yogurt: Tertile 3=OR=**1.40 (95% CI=1.04-1.89)** p-trend=**0.01** Cheese: Tertile 3=OR=**1.80 (95% CI=1.27-2.56)** p-trend=**0.001** Yogurt drink: Tertile 3=OR=1.37 (95% CI=0.98-1.91) p-trend=0.12
Deschasaux-Tanguy et al. (2021) [51]	7,766	Adults, healthy	Food frequency diary evaluated intakes in the previous two years of the following: Total dairy Milk Yogurt Cheese	COVID-19 seroprevalence	Multivariate logistic regression: Total dairy: All COVID cases: OR=**1.19 (95% CI=1.06-1.33)** Symptomatic COVID cases: OR=1.12 (95% CI=0.96-1.31) Asymptomatic COVID cases: OR=**1.29 (95% CI=1.10-1.50)** Milk: OR=**1.15 (95% CI=1.03-1.27)** Yogurt: OR=**1.12 (95% CI=1.00-1.25)** Cheese: OR=0.96 (95% CI=0.84,1.09)
Cameron et al. (2004) [52]	Case: 172 Control: 169	Children, healthy	Food frequency questionnaire evaluated intakes in the preceding two months of the following: Milk Cheese	Clinically confirmed *Campylobacter jejuni* infection	Cheese slices: OR=**0.33 (95% CI=0.51-0.71)** Block cheese: OR=**0.38 (95% CI=0.19-0.76)** Milk (full-cream): OR=**0.53 (95% CI=0.29-0.97)** Reduced-fat milk: Differences NSS (no *p*-value reported) cases vs. control
Yordanov et al. (2017) [53]	294	Adults, healthy	Questionnaire on frequency of yogurt consumption	Seroprevalence of *H. pylori*	All cases: Difference NSS (*p*=0.387) frequent yogurt consumers (≥5 days/week) vs. non-frequent yogurt consumers CagA+ cases: OR=**0.560 (95% CI=0.341-0.921)**

*CagA* Cytotoxin-associated gene A, *NSS *Not statistically significant, *OR *Odds ratio, *SS *Statistically significant

Statistical comparisons that were significant at the *p*=0.05 level are bolded

**Table 5:** Studies of dairy products/probiotics on the natural history of chronic infections (*N*=5)

**Author (Year)**	**Exposure(s) being studied**	**Study population**		**Dairy intervention details**	**Measures of bacterial/viral load**	**Measures of symptoms**	**Measures of quality of life**
		**N**	**Age, gender and health status**				
Ishizaki et al. (2017) [54]	Fermented milk with *L. casei* Shirota	60	Children, HIV-infected	65 mL milk fermented with *L. casei* Shirota daily for 8 weeks	Plasma viral load among HIV positive, without ART: SS decrease (***p*=0.004**) pre- vs. post-intervention Plasma viral load among HIV positive, with ART: Differences NSS (*p*=0.878) pre- vs. post-intervention	NR	NR
Irvine et al. (2010) [55]/ (2011) [56]	Probiotic yogurt with *L. rhamnosus* GR-1 (Fiti)	Exp: 68 Control: 82	Adults, HIV-infected visiting three nutrition programs associated with the West Heads East project	Exp: Persons visiting the sites and consuming 200 mL portions of yogurt supplemented with *L. rhamnosus* GR-1 (Fiti) more than once a week Control: Persons visiting the sites for other nutritional interventions	NR	Proportion self-reporting diarrheal symptoms: SS decrease (***p*=0.05**) exp vs. control Median self-reported days of fever: SS decrease (***p*=0.01**) exp vs. control Proportion self-reporting itching rash: Differences NSS (*p*=0.25) exp. vs. control Proportion self-reporting abnormal/severe stomach pain among ART users: SS decrease (***p*=0.02**) exp vs. control Proportion self-reporting abnormal/ severe nausea and diarrhea among ART users: Differences NSS (*p*=0.20 and 0.10, respectively) exp vs. control	Median self-reported hours able to work: SS increase (***p*=0.01**) exp vs. control Proportion self-reporting moderate/severe impact of GI symptoms on everyday life: SS decrease (***p*=0.03**) exp vs. control
Hummelen et al. (2011) [57]	*Lactobacillus rhamnosus* GR-1 [given in yogurt]	Exp: 55 Control: 56	Adults, HIV-infected	Exp: 125 mL probiotic yogurt fortified with micronutrients and *L. rhamnosus* GR-1 once daily for 4 weeks Control: 125 mL traditional yogurt fortified with micronutrients once daily for 4 weeks	NR	Proportion self-reporting diarrhea: Differences NSS (*p*=0.6) exp vs. control Proportion self-reporting mouth ulcers, coughing, fever, nausea, stomach pain: Differences NSS (*p*-values not reported) exp vs. control	Proportion self-reporting physical energy levels: Differences NSS (*p*-value not reported) exp vs. control Proportion self-reporting ability to perform daily activities: Differences NSS (*p*-value not reported) exp vs. control
Yoon et al. (2019) [58]	*L. paracasei* HP7 and *Glycyrrhiza glabra* (licorice) [given in milk]	Exp: 63 Control: 65	Adults,* H. pylori* infected	Exp: 150 mL of milk fermented with *L. paracasei* HP7 and *Glycyrrhiza glabra* (licorice) daily for 8 weeks Control: 150 mL of placebo drink daily for 8 weeks	Bacterial density on histologic exam: Differences NSS (*p*=0.851) exp vs. control Differences NSS (*p*=0.206) pre- vs. post-treatment among treatment group Differences NSS (*p*=0.182) pre- vs. post-treatment among control group Bacterial density measured by urea breath test: Differences NSS (*p*=0.985) exp vs. control SS decrease (***p*=0.035**) pre- vs post-treatment among treatment group Differences NSS (*p*=0.130) pre- vs post-treatment among control group	Overall gastrointestinal symptoms measured by GSRS: SS decrease (***p*=0.049**) pre- vs. post-treatment among exp group Differences NSS (*p*=0.106) pre- vs. post-treatment among control group	WHOQOL-BREF physical health domain score: SS increase (***p*=0.029**) pre- vs. post-treatment among exp group Differences NSS (*p*=0.347) pre- vs. post-treatment among control group WHOQOL-BREF psychologic, social relationship, and environment domain scores: Differences NSS (*p*=0.684, 0.443, 0.253, respectively) pre- vs. post-treatment among exp group Differences NSS (*p*=0.481, 0.447, 0.697, respectively) pre- vs. post-treatment among control group
Felley et al. (2001) [59]	*L. johnsonii* La 1 [given in milk]	Exp: 26 Control: 27	Adults,* H. pylori* infected	Exp: 180 mL of fermented milk with *L. johnsonii* La 1 twice daily for 3 weeks Control: 180 mL of regular milk twice daily for 3 weeks	Mean bacterial density in antrum: SS decrease (*p*=**0.02**) pre- vs post-treatment among exp group Differences NSS (*p*=0.08) pre- vs. post-treatment among control group Mean bacterial density in corpus: SS decrease (*p*=**0.04**) pre- vs. post-treatment among exp group Differences NSS (*p*=0.12) pre- vs. post-treatment among control group	Mean gastric inflammation in antrum: SS decrease (*p*=**0.02**) pre- vs. post-treatment among exp group Differences NSS (*p*=0.5) pre- vs. post-treatment among control group Mean gastric inflammation in corpus: Differences NSS (*p*=0.2) pre- vs. post-treatment among exp group Differences NSS (*p*=0.8) pre- vs. post-treatment among control group Mean activity of gastric inflammation in antrum: SS decrease (*p*=**0.01**) pre- vs. post-treatment among exp group Differences NSS (*p*=0.6) pre- vs. post-treatment among control group Mean activity of gastric inflammation in corpus: SS decrease (*p*=**0.02**) pre- vs. post-treatment among exp group Differences NSS (*p*=0.3) pre- vs. post-treatment among control group	NR

*ART* Antiretroviral treatment, *GSRS *Gastrointestinal symptom rating scale, *NR *Not reported, *NSS* Not statistically significant, *OR *Odds ratio, *SS *Statistically significant, *WHOQOL-BREF *World Health Organization Quality of Life

Statistical comparisons that were significant at the *p*=0.05 level are bolded

**Table 6:** Studies of dairy proteins on the natural history of infections (*N*=6)

**Author (Year)**	**Exposure(s) being studied**	**Study population**		**Dairy intervention details**	**Measures of viral load**	**Measures of symptoms**
		**N**	**Age, gender and health status**			
Algahtani et al. (2021) [60]	Bovine lactoferrin	Exp 1: 18 Exp 2: 18 Control: 18	Adults, COVID-19 infected (mild to moderate)	Exp 1: 200 mg lactoferrin daily for 7 days Exp 2: 200 mg lactoferrin twice daily for 7 days Control: no treatment	NR	Fever, dry cough, tiredness, diarrhea, headache, and loss of taste and/or smell: Differences NSS (*p*-value=0.802, 0.725, 0.849, 0.763, 0.570, and 0.885, respectively) exp 1, exp 2 vs. control at day 7
Campione et a. (2021) [62]	Bovine lactoferrin	Exp: 32 Control 1: 32 Control 2: 28	Adults, COVID-19 infected (asymptomatic and mild to moderate)	Exp: Liposomal bovine lactoferrin 1 gram, divided into 3 daily oral administrations, or 16 mg divided into 3 daily intranasal administrations for 30 days Control 1: Standard of care regimen for 5-20 days Control 2: No treatment	Mean time to SARS-CoV-2 RNA negativization: SS decrease (***p*<0.001** for both) exp vs. control 1 and exp vs. control 2	
Rosa et al. (2021) [61]	Bovine lactoferrin	121 (82 treated with lactoferrin and 39 untreated)	Adults, COVID-19 infected (asymptomatic and mild to moderate)	Asymptomatic group COVID-19: 200-1,000 mg lactoferrin daily Symptomatic COVID-19: ≥400 mg lactoferrin daily Untreated: Standard-of-care	Median time to SARS-CoV-2 RNA negativization: SS decrease (***p*<0.001**) treated vs. untreated Median time to SARS-CoV-2 RNA negativization, mild to moderate symptoms: SS decrease (***p*<0.001**) treated vs. untreated Median time to SARS-CoV-2 RNA negativization, asymptomatic: Differences NSS (no *p*-value reported) treated vs. untreated Cumulative proportion of SARS-CoV-2 RNA negativization, Kaplan-Meir analysis: SS increase (***p*=0.003**) treated vs. untreated Multiple Cox regression model for RNA negativization: HR=**1.65 (95% CI=1.09-2.25)**	Median time to symptom resolution: Difference NSS (*p*=0.50) treated vs. untreated
Ueno et al. (2006) [63]	Bovine lactoferrin	Exp: 97 Control: 101	Adults, chronic HCV	Exp: 1.8 grams bovine lactoferrin twice daily for 12 weeks Control: Placebo twice daily for 12 weeks	Virological response rate (≥50% decrease in serum HCV RNA at 12 weeks vs. baseline): Differences NSS (*p*-value not reported) exp vs. control	NR
Kaito et al. (2007) [64]	Bovine lactoferrin	Exp: 42 Control: 55	Adults, chronic HCV	Exp: Bovine lactoferrin 3.6 g daily for 8 weeks, followed by lactoferrin, interferon and ribavirin for 24 weeks Control: Interferon and ribavirin for 24 weeks	Mean HCV RNA titer: SS decrease (***p*<0.05**) pre- vs. post-intervention (8 weeks) among exp group Differences NSS (*p*-value not reported) pre- vs. post-intervention (8 weeks) among control group Virological response rate (≥50% decrease in serum HCV RNA): SS increase (***p*<0.05**) exp. vs control at 8 weeks Sustained virological response rate (absence of serum HCV RNA at 24 weeks): SS increase (***p*<0.05**) exp. vs control at 24 weeks among responders	NR
Ishibashi et al. (2005) [65]	Bovine lactoferrin	Exp: 18 Control: 18	Adults, chronic HCV	Exp: 300 mg lactoferrin twice daily for 24 weeks, interferon dose of 6 million units daily for 2 weeks followed by three times per week for 22 weeks, and 600-800 mg ribavirin twice daily for 24 weeks Control: Placebo twice daily for 24 weeks, interferon dose of 6 million units daily for 2 weeks followed by three times per week for 22 weeks, and 600-800 mg ribavirin twice daily for 24 weeks	Sustained virological response rate (absence of serum HCV RNA at 24 weeks): Differences NSS (*p*=0.7) exp vs. control at 24 weeks	NR

*HCV* Hepatitis C virus, *HR* Hazard ratio, *NR* Not reported, *NSS* Not statistically significant, *OR* Odds ratio, *SS* Statistically significant

### Risk of bias assessments

Risk of bias (RoB) assessment was evaluated using the Academy of Nutrition and Dietetics Quality Criteria Checklist [66], which was specifically designed for nutritional studies. This checklist collects yes, no, not available (NA), or unclear responses to 10 validity questions to assess various domains where bias can arise in a study (e.g., inclusion and exclusion criteria, withdrawal, data collection, data analysis, and conflicts of interest). RoB assessment was conducted by one reviewer; the results were reviewed independently by a second reviewer for complete equality control. A senior reviewer resolved any conflicts and finalized the RoB results. Study quality was determined as positive quality, neutral quality, or negative quality, depending upon the scoring results from the domains (Supplemental Table 2, Additional file 3).

### Data synthesis

Qualitative synthesis was conducted, as meta-analysis could not be performed due to the heterogeneous nature of the dairy exposures and reported outcomes. Results are summarized below by outcome, including the incidence, duration, and severity of infections (Tables 2, 3 and 4) and the natural history of infectious diseases (Tables 5 and 6). Within each of these outcomes, studies were summarized by the exposure/intervention, including whole dairy products, a particular probiotic added to a dairy product, dairy proteins, and dietary intake of dairy. Milk products were separated by traditional and fermented milk, and yogurt was separated by traditional and probiotic yogurts. This level of exposure and comparison group detail was considered to evaluate what component of the dairy product could be beneficial – the cumulative matrix of the whole dairy product, traditional yogurt ferments, particular probiotic strains delivered in dairy, and/or proteins. Studies were also summarized by bacterial strain, as beneficial effects may be strain specific. Additional effect modifiers that were considered in the qualitative synthesis included the age and health status of the population.

## Results

### Article identification

**Fig. 1:**
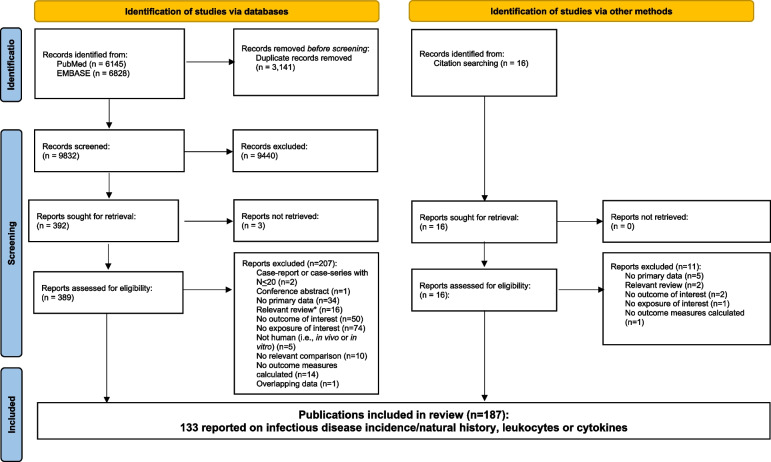
PRISMA flow diagram. *Four of these review articles were not searched for additional references because the topic of the article was bovine colostrum and/or hyperimmune milk. Source of flow diagram template: Page et al. 2021 [17]

Figure 1 displays the PRISMA flow diagram detailing study inclusion/exclusion at each stage of review. The database searches yielded 12,973 hits. After de-duplication across databases, 9,832 abstracts were screened, 389 of which were identified as potentially relevant and flagged for full-text review. After reviewing the full-text articles, 207 articles were excluded for the following reasons: 74 did not have any exposures of interest, 50 had no outcomes of interest, 34 had no primary data (i.e., meta-analyses, opinion pieces, or reviews), 16 were relevant reviews, 14 had no effect measures calculated or statistical testing, 10 had no relevant comparison group, 5 were an *in vitro* or *in vivo* study, 2 were case reports or case-series with less than 20 patients, 1 was a conference abstract, and 1 publication was excluded because its primary data was included in another publication. Sixteen relevant reviews were identified, and 5 additional studies were identified by examining their reference lists. Thus, 187 publications meeting the pre-defined PICOS criteria were included in the overall SLR.

Among the 187 publications, 133 relevant publications were identified, reporting on 128 unique studies; many of these studies reported on more than one outcome relevant to this SLR. Our review identified 34 studies specifically examining the incidence, duration, and/or severity of acute infectious diseases (Tables 2, 3, and 4) and 11 studies on the natural history of infectious diseases (Tables 5 and 6). Our review also identified 76 studies of leukocyte levels and measures of leukocyte activity, proliferation, cytotoxicity and phagocytosis (Supplemental Table 3, Additional file 3) and 47 studies of cytokine production (Supplemental Table 4, Additional file 3). Five instances were identified where two publications reported results from the same study [28, 55, 56, 67–73]; however, both publications were included because they provided unique data. Future publications will discuss other immune-related outcomes.

### Characteristics of included studies on the incidence and natural history of infectious diseases (*N*=45)

Table 1 presents the characteristics of the 45 included studies on the incidence and natural history of infectious diseases. Of the 45 studies, 31 (69%) were given a positive quality rating, 12 (27%) a neutral rating, and 2 (4%) a negative rating. Thirty-eight (84%) were clinical trials, 5 were cohort studies, 1 was a case-control study, and 1 was cross-sectional. These studies were conducted in diverse locations, with 44% in Asia, 33% in Europe, 7% in North America (all US), 7% in Africa, 4% in South America, and 4% in Australia. The time period of study enrollment/follow-up ranged from 2000-2021. The most common exposure being investigated was a probiotic added to a milk or yogurt product (*N*=16), followed by whey proteins (*N*=13). The infectious disease under investigation varied significantly and included acute infections such as respiratory infections, influenza, and COVID-19 and chronic infections such as HIV and hepatitis C.

### Incidence of acute infection (Tables 2, 3 and 4)

#### Whole dairy products (*N*=23, Table 2)

Twenty-three controlled, clinical trials were identified that administered whole dairy products in the experimental group and followed the study population prospectively for acute infectious disease incidence (Table 2). The comparison group varied widely between studies, with studies designed to examine the cumulative exposures associated with milk [20], fermented milk [21–25], traditional yogurt [26–28], and probiotic yogurt [28, 29] ingestion and other studies designed to examine the impact of adding a particular probiotic to the fermentation process of milk or yogurt drinks [30–42]. The infectious disease under consideration also varied and included broad categories of infections with a self-limited duration, including “winter infections” [21] and CIDs [24, 25, 31–33]. Some studies specifically evaluated respiratory tract infections (RTI) [34, 36, 38] and symptoms [41], upper respiratory infections (URTI) [20, 29] and symptoms [35], the common cold and influenza [26–28, 30, 39, 40], and norovirus gastroenteritis [22]. The method for evaluating disease incidence also varied from clinical verification of incident cases to disease definitions based solely on questionnaire data. The majority (*N*=15 or 65%) of these clinical trials were randomized and double-blinded [23–25, 30–36, 38–42]. The most common study locations were Japan and Italy (*N*=7 and *N*=6, respectively), with two studies conducted in North America (Table 1). Nine of these studies were conducted in healthy, adult populations [20, 21, 28–30, 37, 39–41], five were conducted among children attending daycare centers/schools [24, 25, 33, 38, 42], and five were conducted among elderly persons, both free-living [27, 32] and institutionalized [22, 23, 36]. Other potentially immune challenged populations were considered, including two studies of healthcare/shift workers [26, 31] and two studies of persons undergoing intense physical training [34, 35]. The vast majority (*N*=21, or 91%) of the studies were categorized as positive (*N*=15) or neutral (*N*=6) quality on RoB assessment (Table 1).

Only one study was identified that evaluated the impact of non-fermented milk (Tables 1 and 2). The average number of incident URTI cases was significantly lower (*p*<0.01) among Japanese adults consuming milk during weekly physical exercise for one year (compared to those consuming a sports drink), and a significant inverse correlation (*p*=0.03) between total dairy consumption and URTI severity was reported [20]. The study was limited, however, by a small sample size (*N*=13), a lack of blinding, and URTI diagnosis based on self-report [20].

Five clinical trials evaluated the cumulative impact of fermented milk, which included traditional ferments and ferments from various additional *Lactobacillus* strains (Tables 1 and 2). Mixed findings were reported. Two relatively large trials of Italian school children (*N*=146 and 268, respectively) reported a significantly lower proportion of clinically verified CID among children given 150 mL of milk fermented with *L. paracasei* CBA l74 daily for three months compared to children given a drink with a similar energy content, with significant incidence rate ratios (IRR) of 0.64 (95% CI=0.42-0.98) [24] and 0.36 (95% CI=0.29-0.44) [25]. A large trial (*N*=360) of healthy Italian adults given Actimel® (fermented milk with traditional ferments and *L. casei* DN-114 001) twice daily for three months found no difference in the cumulative incidence of clinically verified winter infections compared to no intervention [21], although the study was categorized as negative on RoB assessment due to the lack of details provided on the exposure, intervention and withdrawal. A study of elderly hospitalized Japanese patients found no reduction in the cumulative incidence of norovirus gastroenteritis associated with the daily ingestion of fermented milk with *L. casei* Shirota, compared to no treatment, during their hospital stay [22]. Despite mixed findings on disease incidence, the studies of fermented milk did, however, report consistent decreases in the duration of infections (winter infections, *p*=0.02 [21]; infections requiring antibiotics, *p*=0.05 [23]), the duration of symptoms ([fever associated with norovirus gastroenteritis, *p*=0.03 [22]), and measures of disease severity (CID, *p*=0.02 [24]; CID, *p*<0.001 [25]), although the data were collected largely in an open-label setting [21–23].

Mixed results were also reported on the benefits of traditional and probiotic yogurt (Tables 1 and 2). Two open-label trials in Japan examined the impact of daily ingestion of a traditional yogurt (Meiji Probio Yogurt R1® with *L. delbrueckii* ssp. *bulgaricus*) on the incidence of the common cold and influenza. One trial randomized female healthcare workers to the yogurt drink (*N*=479) or no supplementation (*N*=482) for 16 weeks [26]. There was no statistically significant difference in the cumulative self-report of a physician diagnosis of influenza or common cold between the yogurt and control groups, nor were there any statistically significant differences in influenza or common cold cumulative incidence in Kaplan-Meir analysis. The second trial randomized elderly Japanese persons to Meiji Probio Yogurt R1® or milk in two separate study locations for 8 (Fungata) and 12 weeks (Arita); a reduced odds of cold or influenza was observed when the two sites were meta-analyzed (OR=0.39, *p*=0.02) [27]. In a crossover trial of 30 US adults, no statistically significant difference was reported between the number of influenza and cold cases the month prior to the study compared to during treatment (treatment included traditional yogurt smoothie, a yogurt smoothie with added *Bifidobacterium (B.) animalis* subsp. *lactis* [BB-12] pre-fermentation, and a yogurt smoothie with BB-12 added post-fermentation); subjects consuming traditional yogurt smoothie and BB-12 added pre-fermentation experienced significantly fewer number of days with cold/flu symptoms (*p*<0.05 and *p*<0.01, respectively) [28]. Finally, in a study comparing probiotic yogurt supplemented with *L. paracasei* N1115 to no yogurt for 12 weeks among Chinese adults, a reduced risk of URTI events based on self-reported symptoms was found (RR=0.55, 95% CI=0.31-0.97) [29], similar in magnitude to the risk reductions for CID observed with *L. paracasei* fermented milk described above [24, 25].

The remaining thirteen clinical trials were designed to evaluate whether the addition of probiotic(s) to milk or yogurt drinks influenced the risk of infectious diseases [30–42]. One study investigated a probiotic from the genus *Bifidobacterium* [30], one study investigated a probiotic from the genus the genus *Lactococcus* [39], and the remainder of the studies investigated a probiotic from the genus *Lactobacillus*, with seven investigating *L. casei* [31, 32, 34–37, 74], one investigating *L. johnsonii* [23], one investigating *L. rhamnosus* GG [38], and three investigating a combination of *Lactobacillus* strains [40–42] (Tables 1 and 2). These studies are summarized below by bacterial genus and strain.

In the one identified trial of the probiotic species *Bifidobacterium*, 136 Chinese adults were randomized to 250 g of a yogurt supplemented with *B. animalis* subsp. *lactis* B1-04 (Qingrun®) or a control yogurt daily for three months (Tables 1 and 2). The supplemented yogurt was associated with a statistically significant approximately 60-70% reduction in the incidence of common cold and influenza-like illness (OR=0.38 [95% CI=0.17-0.81] and 0.32 [95% CI=0.11-0.97], respectively). The supplemented yogurt was also associated with a statistically significant reduction in URTI duration (*p*<0.0001) and severity (*p*<0.0001) [30], similar to the findings from the study of BB-12 supplemented yogurt smoothies [28].

Three large, double-blind clinical trials randomized study participants (*N*=1,000 German shift works, *N*=972 elderly Germans, and *N*=638 US children) to 200 mL of a dairy drink (Europe: Actimel®; US: DanActive®) fermented with *L. casei* DN-114 or a non-fermented dairy drink and followed them for the incidence of CID for three months, with mixed findings (Tables 1 and 2). The study of shift workers reported no difference in the cumulative number of all CIDs by Poisson regression, but a statistically significant reduced odds of CID (OR=0.70, 95% CI=0.54-0.90); a significant reduction in the cumulative duration of fever was also found (*p*=0.02), but no difference was found for other measures of disease severity/duration [31]. Similarly, the study of US children reported a reduced incidence of CID associated with DanActive® (IRR=0.81, 95% CI=0.65-0.99), but no impact on symptom duration or severity [33]. In contrast, the study of elderly Germans found no difference in the cumulative number of CIDs or the mean CID rate, but statistically significant improvements in measures of disease duration [32]. The authors cited the low number of observed events as a possible explanation for the lack of an association between the fermented drink and CID incidence in this study. An additional study of Actimel® was conducted in 47 male Italian cadets; 300 mL was given daily for one month during commando training and no difference was reported in the cumulative number of persons with RTI, the incidence of RTI or the duration of symptoms [34].

Three clinical trials of milk fermented with *L. casei* Shirota (Yakult®), compared to a non-fermented milk, reported trends toward an improvement in respiratory tract illnesses and symptoms (Tables 1 and 2). The consumption of 80 g of Yakult® 30 days prior to running a marathon was associated with a reduced, but not statistically significant (*p*=0.08), number of persons reporting upper respiratory symptoms after a marathon in Brazil [35]. A large (*N*=773) clinical trial of clinically verified RTI among elderly persons in Belgian nursing homes found no difference in the number of participants with at least one day of RTI symptoms, but a trend toward a reduced odds of developing a severe RTI in logistic regression modeling (OR=0.592, 95% CI=0.335-1.049) [36]. No difference in the duration of symptoms was found in either of these Yakult® trials [35, 36]. Finally, among a healthy population of adults in Japan, the daily consumption of Yakult® for 12 weeks was associated with a statistically significant reduction in the cumulative proportion of patients with an incident URTI (*p*=0.002) and cold (*p*=0.005) event, but not an influenza event; statistically significant reductions in the duration of each URTI episode (*p*=0.002) and the cumulative days with URTI symptoms (*p*=0.001) was also observed [37]. The authors suggested the age of the study participants may explain the conflicting results from the Yakult® studies, i.e., the older patients may be less responsive to the immune modulating effects of the yogurt drink [37].

In a study of Japanese children attending daycare who were randomized to *L. rhamnosus* GG fermented milk (*N*=282) or non-fermented milk (*N*=289) three times daily for seven months, a reduced odds of RTI was observed with treatment, but it failed to meet statistical significance (age-adjusted OR=0.75, 95% CI=0.53-1.09) [38]. Another Japanese study randomized healthy adults to a yogurt drink with *Lactococcus lactis* ssp. *lactis* JCM5808 daily for 12 weeks or a non-fermented yogurt drink and found no difference in the cumulative incidence of influenza or cold cases, but a statistically significant decrease in the duration of some symptoms (cough and feverishness, *p*<0.001 for each and severe sore throat, *p*=0.01) [39].

Three additional trials compared dairy products with combinations of added *Lactobacillus* species to a non-fermented control group with mixed findings (Tables 1 and 2). Daily consumption of a yogurt drink fermented with *L. paracasei, L. casei* 431 and *L. fermentium* PCC for 12 weeks was associated with a statistically significant reduction in the proportion of Chinese adults with URTI (*p*=0.002) and influenza-like illness with a fever (*p*=0.03), as well as a reduction in URTI duration (*p*<0.001) and various measures of severity [40]. A study of 200 mL daily milk ingestion with *L. rhamnosus* IMC 501 and *L. paracasei* IMC 502 for four weeks found no association with the self-report of respiratory symptoms, but was limited by a small size (*N*=10) and categorized as “negative” in RoB assessment (Table 1) [41]. In a study of low socioeconomic status (SES) Brazilian children, 140 children were randomized to milk fermented with *L. casei* CRL431 and *L. acidophilus* or non-fermented milk and no difference was found between the number of patients with URTI, gastroenteritis, varicella, or pneumonia [42].

#### Dairy proteins (*N*=7, Table 3)

Seven studies investigated the potential impact of dairy protein supplements on the incidence of acute infectious diseases (Tables 1 and 3). One study evaluated hydrolyzed whey protein [43] and another study evaluated a combination of the immunoglobulin rich fraction from whey protein and bovine lactoferrin [44]; no studies of casein protein were identified. The remaining five studies evaluated bovine lactoferrin [45–49]. The studies investigated a range of outcomes, including respiratory tract infections [44–46], bacteremia [43], and sepsis [47–49]. The studies were placebo-controlled, randomized, double-blind clinical trials, except for one retrospective cohort [43]. Three of the studies were conducted in adult populations [43–45] and four were conducted in infants [46–48, 75]. All of the studies were categorized as positive (*N*=6) or neutral (*N*=1) quality on RoB assessment (Table 1).

Three studies of whey protein supplements suggested that this milk protein may reduce the incidence and burden of common infectious diseases, although the evidence base is small and the trials were diverse in the age of the study population, dosing schedule and outcome. A trial of 105 Australian adults with recurrent colds reported that taking a combination of the immunoglobulin rich fraction from whey protein and lactoferrin for three months significantly reduced self-reported cold events (*p*<0.001) and symptoms over that time (*p*<0.05), compared to placebo, but had no statistically significant impact on cold duration [44]. A trial of 209 Japanese adults reported no significant differences in the prevalence or number of episodes of infectious diseases (the majority of which were summer colds) between participants given placebo, 200 mg or 600 mg of bovine lactoferrin daily for 12 weeks, but reported significant reductions in the duration of all infectious diseases (*p*=0.05 and 0.01 for 200 mg and 600 mg dosing, respectively), with a significant dose-response trend (*p*=0.01). The duration of common colds was also significantly shorter (*p*=0.04) among participants given 600 mg lactoferrin, but a similar pattern was not observed for the other infectious diseases, including gastroenteritis, cold sores and styes [45]. Finally, 52 infants were given either regular cow milk based formula or formula with added bovine lactoferrin for one year in a US trial; while the lactoferrin supplemented formula was associated with a significant (*p*<0.05) reduction in the average number of lower respiratory tract infections (LRTI) per infant-year, similar associations were not observed for URTI or acute otitis media (AOM) and no differences in disease duration were found [46].

Although the evidence base is small (*N*=4), studies suggest whey protein supplements reduce the risk of bacteremia/sepsis in adults and infants. A retrospective cohort of 76 Japanese adults receiving a liver transplant found that patients administered hydrolyzed whey peptides as part of their enteral nutrition post-transplant had a statistically significant reduction (*p*=0.002) in the occurrence of bacteriemia, compared to patients that received standard enteral nutrition [43]. Three studies enrolled low birth weight/pre-term hospitalized infants, provided 100-250 mg of bovine lactoferrin daily, and followed them for sepsis. The largest study of infants randomized Italian participants to 100 mg bovine lactoferrin (*N*=153), 100 mg bovine lactoferrin with *L. rhamnosus* GG (*N*=151), or placebo (*N*=168) for the first 30 days of life; a multivariate logistic regression analysis for late-onset sepsis reported ORs of 0.32 (95% CI=0.14-0.77) and 0.21 (95% CI=0.08-0.55) for bovine lactoferrin and bovine lactoferrin/*L. rhamnosus* GG treatment, respectively [49]. These late-onset sepsis events included bacterial and fungal infections, and a statistically significant reduction in the risk of sepsis was reported for bacterial episodes alone (no *p*-values were reported). An Indian trial (*N*=130) administered either bovine lactoferrin (with weight-based dosing) or placebo for the first 30 days of life and found a significant reduction in all sepsis events (*p*=0.001) and sepsis-attributable mortality (0.03) [47]. Similarly, in a trial of low-birthweight or pre-term infants in Turkey, a significant reduction (*p*=0.01) in the rate of sepsis was found in the treatment group (*N*=25, 200 mg bovine lactoferrin daily), compared to placebo (*N*=22) [48].

#### Dietary patterns involving dairy (*N*=4, Table 4)

Four studies reported associations between infectious diseases and dairy exposures measured by responses on food frequency questionnaires (Tables 1 and 4). Two large prospective cohort studies (one conducted in Iran and the other in France) measured the association between estimates of dairy, milk, yogurt, and cheese intake in the years prior to the pandemic and the seroprevalence of COVID-19 with multivariate logistic regression models. Weak, statistically significant positive associations were observed for estimates of high-fat dairy (OR=1.40, 95% CI=1.09-1.92), high-fat milk (OR=1.54, 95% CI=1.20-1.97) and yogurt (OR=1.40, 95% CI=1.04-1.89) intake in the Iranian cohort [50], and for total dairy (OR=1.19, 95% CI=1.06-1.33), milk (OR=1.15, 95% CI=1.03-1.27) and yogurt (OR=1.12, 95% CI=1.00-1.25) intake in the French cohort [51]. A statistically significant 50% reduction in the odds of COVID-19 seropositivity (OR=0.51, 95% CI=0.37-0.69) was observed with low-fat dairy products in the Iranian cohort [50]. In addition, a case-control study of Australian children reported cheese and full-cream milk consumption had significant protective effects (*p*=0.003 and 0.04, respectively) on *Campylobacter jejuni* infection [52] and a cohort study of Bulgarian adults reported frequent yogurt consumption had a significant protective effect (*p*=0.05) on *Heliobacter (H.) pylori* cytotoxin-associated gene A (CagA) seropositivity [53].

### Natural history of infectious diseases (*N*=12, Tables 5 and 6)

#### Whole dairy products (*N*=5, Table 5)

Five studies were identified that evaluated the effect of whole dairy products/probiotics on a wide variety of outcomes related to the natural history of chronic infectious diseases (Tables 1 and 5). Three studies investigated the effect of whole dairy products, including fermented milk in children [54], probiotic yogurt in adults [55, 56] and a probiotic delivered in yogurt [57], on the natural history of HIV, with mixed findings. In a clinical trial of 60 Vietnamese children administered 65 mL milk fermented with *L. casei* Shirota daily, plasma viral load was found to decrease after 8 weeks compared to baseline (*p*=0.004) [54]. In a retrospective cohort of HIV-infected adults in Tanzania visiting a network of community-based nutritional intervention sites, daily ingestion of yogurt containing *L. rhamnosus* GR-1 was found to improve symptoms (diarrhea, *p*=0.05 and fever, *p*=0.01) and quality of life (ability to work, *p*=0.01 and impact of GI symptoms on daily life activities), compared to a group of HIV infected adults visiting the sites for other nutritional interventions [55, 56]. Contrary to these findings, however, no difference in symptoms, physical energy levels and the ability to perform daily activities was reported between HIV-infected adults given 125 mL yogurt with *L. rhamnosus* GR-1 (*N*=55) daily for 4 weeks, compared to HIV-infected adults given the same regimen of traditional yogurt (*N*=56) in a randomized, placebo-controlled, double-blind clinical trial in Tanzania [57].

Two randomized, placebo-controlled and blinded trials evaluated *H. pylori*-infected adults, the results of which suggest probiotics may help eradicate and improve symptoms of *H. pylori* infections [58, 59]. In a trial in South Korea, a statistically significant decrease (*p*=0.04) in the urea breath test was found when baseline levels were compared to those observed after 8 weeks of daily 150 mL consumption of *L. paracasei* HP7 fermented milk; no differences were found, however, when the treatment arm (*N*=65) and control arm (*N*=63, 150 mL daily consumption of regular milk) were compared. Some measures of symptoms were improved in the treatment group (gastrointestinal symptoms, *p*=0.05 and the physical health domain score of the World Health Organization Quality of Life [WHOQOL]-BREF, *p*=0.03) [58]. The other clinical trial of *H. pylori* infected adults reported 360 mL daily consumption of *L. johnsonii* La1 fermented milk for three weeks was associated with decreased bacterial density in the antrum and corpus (*p*=0.02 and *p*=0.04, respectively), as well as a decrease in gastric inflammation scores (*p*=0.02 for the antrum) and activity of gastric inflammation scores (*p*=0.01 and *p*=0.02 for the antrum and corpus), compared to pre-intervention levels; similar differences were not found in the control group of regular milk consumption [59].

#### Dairy proteins (*N*=6, Table 6)

Six studies investigated the impact of bovine lactoferrin on the natural history of infections in adults, including COVID-19 (*N*=3) [60–62] and hepatitis C (*N*=3) [63–65] (Tables 1 and 6).

The three studies of COVID-19 patients recruited asymptomatic and mild/moderate COVID-19 patients during the early stage of the pandemic in 2020-2021 [60–62]. Although the evidence is limited by the small number of studies and recruited patients, the studies suggested lactoferrin reduced the time to SARS-CoV-2 seroconversion but did not affect symptom resolution. In a clinical trial in Italy, patients were given liposomal bovine lactoferrin 1 g orally or 16 mg intranasally daily (*N*=32), standard of care treatment (*N*=32) or no COVID-19 treatment (*N*=28); the patients receiving bovine lactoferrin had a statistically significant shorter mean time to achieving a SARS-CoV-2 RNA negative test (mean=14.25 days), compared to the standard of care treatment (mean=27.13 days, *p*<0.001) and no treatment groups (mean=32.61 days, *p*<0.001) [62]. A similar finding was observed in another Italian study; a retrospective cohort of 121 COVID-19 patients reported the median time to a SARS-CoV-2 RNA negative test was statistically significantly (*p*<0.001) shorter in those treated with bovine lactoferrin (median=15 days) compared to standard of care treatment (median=24 days) [61]. In a multivariate Cox regression model adjusting for other predictors of SARS-CoV-2 RNA negativization, a HR of 1.65 (95% CI=1.09-2.25) was reported for bovine lactoferrin. No statistically significant difference in the median time to symptom resolution was found in this study [61]. Similarly, a clinical trial in Egypt found no statistically significant difference in the number of participants reporting clinical symptoms of COVID-19 in the treatment groups (*N*=36) compared to the control group (*N*=18) after seven days of treatment with 200-400 mg bovine lactoferrin [60].

Three trials of bovine lactoferrin among chronic hepatitis C virus (HCV) patients were identified. The studies were randomized, double-blind, placebo-controlled trials conducted in Japan that evaluated the impact of 600-7200 mg of bovine lactoferrin for 12-24 weeks, with mixed findings [63–65]. A trial of 3600 mg bovine lactoferrin daily for 12 weeks (*N*=97), compared to placebo treatment (*N*=101), reported no difference in the virological response rate (≥50% decrease in serum HCV RNA) [64]. Likewise, a trial of 600 mg bovine lactoferrin daily with standard HCV treatment for 24 weeks (*N*=18), compared to standard HCV treatment alone (*N*=18), reported no difference in the sustained virological response rate (absence of serum HCV RNA) [63, 65]. In contrast, a statistically significant increase (*p*<0.05) in the virological response rate after 8 weeks of bovine lactoferrin monotherapy (*N*=42), compared to HCV standard therapy (*N*=55), was reported. Furthermore, among the patients responding at 8 weeks in this trial, a statistically significant increase in the sustained virological response rate was observed after 24 weeks of therapy (bovine lactoferrin + standard HCV therapy vs. standard HCV therapy). This trial also found a statistically significant reduction (*p*<0.05) in HCV RNA titers at 8 weeks compared to baseline among the patients in the intervention group [64].

#### Leukocytes (*N*=76), Supplemental Table 3

**Fig. 2:**
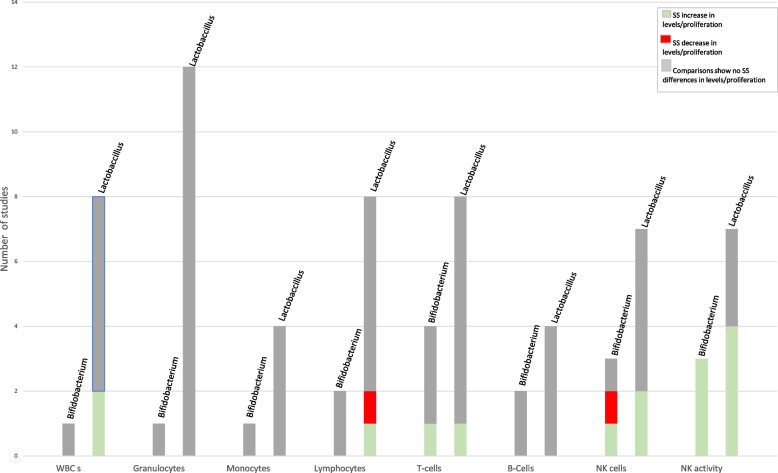
Studies of probiotics administered on leukocyte levels/proliferation/activity, by probiotic strain (*N*=28)

**Fig. 3:**
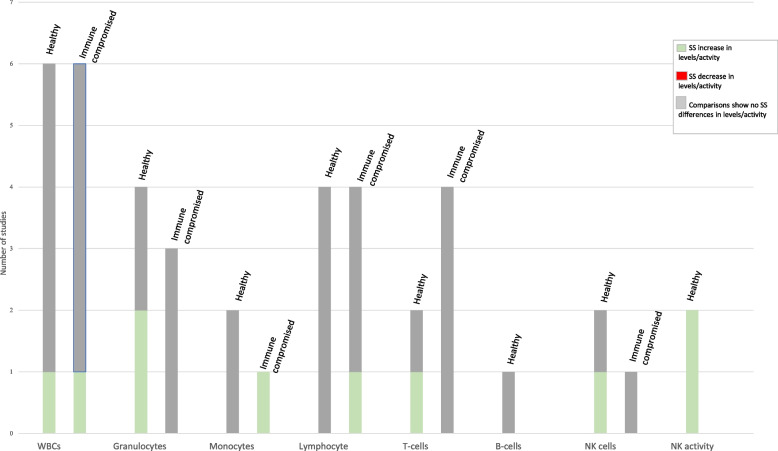
Studies of milk proteins on leukocyte levels/proliferation/activity, by health status (*N*=19)

Supplemental Table 3 describes studies measuring the influence of dairy products and/or their components on levels of white blood cells and their components (i.e., granulocytes including neutrophils, basophils and eosinophils; monocytes; and lymphocytes, including T-cells, B-cells and natural killer [NK] cells). Other *in vitro* measures of immune function were identified, including studies of neutrophil activity, lymphocyte proliferation/activation/transformation, NK cell activity/function/cytotoxicity, and the phagocytic activity, tumoricidal activity and oxidative burst capacity of leukocytes. Additional file 2 provides detailed summaries of the evidence. The studies of leukocytes and probiotics and dairy proteins are summarized in Figs. 2 and 3, respectively. No consistent changes in leukocyte levels and function were observed for any type of whole dairy product or their components. Probiotics and dairy proteins appear to enhance NK cell levels/activity and the phagocytic process in a larger proportion of studies with these outcomes (Figs. 2 and 3). Isolated responses were not consistent across populations, however, and the clinical relevance of these biomarkers of immune response is not clear.

#### Cytokines (*N*=47), Supplemental Table 4

Supplemental Table 4 describes studies that evaluated the effect of dairy products or their components on cytokine production, including the ILs of interest, interferons (IFN), transforming growth factor (TGF)-β, and chemokines of interest. Additional file 2 provides detailed summaries of the evidence. Overall, the results of these studies were conflicting, with most studies reporting no significant impact of dairy products (including whole dairy products, probiotics specifically, and dairy proteins) on cytokine production. Interpretation of results is challenging as biological or clinical relevance was not considered.

## Discussion

Based on this SLR, the cumulative available evidence suggests daily ingestion of dairy products fermented with probiotics from the genus *Lactobacillus* for ~1-3 months may reduce the risk of acquiring common infectious diseases (in particular URTI, cold and influenza) and improve the duration/severity of these diseases. The evidence base is suggestive, but findings are difficult to reconcile because of heterogeneity in the age/health status of the study population, the bacterial strains administered, and the statistics measured. Some studies that observed no difference in infectious disease incidence between treatment and control groups cited the low number of observed incident events in the study population [32, 39] or an overall inadequate sample size [26, 28] as possible explanations for their negative findings. Of note, a recent meta-analysis on the effect of probiotic fermented dairy products on the incidence of RTIs combined studies published through October 2020, thus addressing any issues with insufficient sample size/power [15]. In this meta-analysis, consumption of probiotic fermented dairy products had a significant protective effect against RTIs in the overall analysis (RR=0.81, 95% CI=0.74-0.89) and separately in children (RR=0.82, 95% CI=0.73-0.93), adults (RR=0.81, 95% CI=0.66-1.00) and elderly populations (RR=0.78, 95% CI=0.61-0.98). The benefit was restricted to *Lactobacillus* supplemented products (RR=0.81, 95% CI=0.74-0.90), although only two studies were available on *Bifidobacterium*. Disease-specific analyses showed benefits for URTI, pneumonia and the common cold, but marginal benefits for LRTI. A beneficial role for probiotic fermented dairy products in the prevention of acute infections is consistent with the results of clinical trials of probiotics given in power or pill form; in a recent meta-analysis of studies evaluating the impact of probiotics given in any form on the incidence of URTIs, a combined RR of 0.76 (95% CI=0.67-0.87) was reported for at least one URTI event, with low-certainty evidence [76].

Nearly all studies in our SLR that found no association between probiotic fermented products and the incidence of common infectious diseases reported improvements in some measure of disease severity/symptoms/duration [21, 22, 28, 32, 34, 38, 39], suggesting that even if the immune modulating effect of probiotics are not significant enough to prevent illness, they may still improve the course of disease. These findings are particularly relevant for older populations that are known to have age-related decrements in immune function and a higher incidence of infection, burden of disease, and more severe complications (e.g., with influenza [77]). The incorporation of fermented dairy products into residential elderly settings may be an easy and potentially impactful nutritional intervention to slow the spread and impact of infectious diseases in these settings. While the USDA Dietary Guidelines do not specifically provide a recommendation for fermented dairy products, adults may consider incorporating fermented products into the recommended 3 cups of dairy per day [8].

A small group of studies (*N*=3) evaluated whether lactoferrin monotherapy (200-600 mg daily) can reduce the incidence and burden of common infectious diseases, with mixed results [44–46]. Findings from the three studies of lactoferrin as an enteral supplement to pre-term infants to prevent sepsis were also mixed, which may be due to variability in iron saturation, the route of administration and the dosing schedule [47, 48, 75]. A recent SLR and meta-analysis of lactoferrin supplementation for late-onset sepsis in preterm infants reported a combined RR of 0.82 (95% CI=0.74-0.91) with low-quality evidence [14]. Thus, nutritional intervention with lactoferrin may be a promising strategy to boost human lactoferrin from mother’s milk and prevent infections in infants, although larger and more detailed analyses are required. This intervention may not be directly applicable to dairy products, however, as the concentration of lactoferrin is lower in bovine milk (around 25-75 mg in a glass of milk [78]) and dairy products are not recommended until six months of age.

Two cohorts reported COVID-19 seropositive patients were significantly more likely to report a higher intake of dairy products (in particular high-fat dairy products), compared to COVID seronegative patients [50, 51]. While control for confounding variables was attempted in these studies, residual confounding cannot be ruled out and would be consistent with the weak associations observed (OR=~1.1-1.5). Additional studies are recommended to reconcile these findings, with disease measurement based on clinically confirmed incident infections and measures of verified exposures linked more closely in time to disease incidence.

Our SLR identified a wide variety of potential applications for dairy products/components to improve the natural history of infectious diseases, likely due to the antimicrobial nature of lactoferrin and probiotics, although the available evidence in each research area is small and further research is required. Clinical trials suggest virological/bacteriological burden is reduced with lactoferrin (for COVID-19 [61, 62] and HCV [63–65]) and with probiotic [for *H. pylori* [59] and HIV [54]) treatment; symptoms of these conditions were also reduced with nutritional intervention in some studies [55, 56, 58]. Among persons with HIV/AIDS, the gut-associated lymphoid tissue is a major site of HIV replication and, therefore, represents a vulnerability to these patients, including the development of opportunistic infections. Probiotics can reinforce mucosal barrier function in the gastrointestinal system and modulate immune responses in the intestinal epithelium to improve outcomes in persons with HIV/AIDS. Food based interventions, such as probiotic yogurt, could help delay the progression of HIV/AIDs, particularly in populations with limited access to anti-retroviral treatment. Our review also found that probiotics may be useful to patients with *H. pylori* infection, a common bacterium that colonizes the gastric epithelium and increases the risk for stomach cancer; in addition to supporting gut health in the context of antibiotic treatment for *H. pylori* elimination, the evidence suggests *Lactobacillus* strains may be bactericidal in the gut and have an independent suppressive effect on *H. pylori* [58, 59]. The significant reduction in the time to COVID-19 negativization in two Italian studies suggests that lactoferrin improves viral clearance [9, 62], but the relevance of this research to dairy products is unknown. This finding should be confirmed in future studies, as a shorter time to COVID-19 seroconversion could limit the spread of infection.

One proposed mechanism for these observed effects is a modulation of the immune system by dairy product components. No consistent changes in white blood cells or cytokine production were observed for any type of whole dairy product or their components, among healthy and immune compromised populations (Supplemental Table 3, Supplemental Table 4, Figs. 2 and 3). Probiotics appeared to enhance natural killer cell levels/activity and the phagocytic process in a larger proportion of studies with these outcomes [79–84], suggesting this mechanism could play a key role in the reduction and/or burden of infections. Isolated responses were not consistent across populations, however, and the clinical relevance of these biomarkers of immune response is not clear. Limitations of this group of studies include small sample sizes, varied methods for measuring these biomarkers, a short intervention duration, lack of adjustment for multiple comparisons, and lack of an appropriate control group. Future clinical trials should continue to quantify biomarkers of immune function concurrently with disease incidence as measured by discrete antibody titers.

The main strength of this SLR is that the scope was broad, with few restrictions on exposures, outcomes, or study population characteristics. As such, this SLR provides a comprehensive scoping of the available evidence. While other reviews and meta-analyses provided a summary of specific dairy products and particular components, this SLR summarized all dairy products, including traditional and fermented products, and dairy proteins. Furthermore, this SLR included data on the impact of dairy products/components on leukocytes and cytokines, to potentially connect the epidemiologic findings with a mechanism. The quality of the evidence base is relatively strong, with the 96% of the studies classified as “positive” or “neutral” based on RoB assessment. This SLR also identified largely randomized, double-blind, controlled trials which are considered to be one of the strongest forms of epidemiologic evidence. Another strength of this review is that we thoroughly evaluated the component of exposure under study to differentiate the impact of the full dairy matrix versus the impact of specific probiotics strains; this approach has not been used in the previous reviews of this topic and allows for a better understanding of which dairy component may be bioactive.

While this SLR suggests a beneficial role for dairy in the incidence and natural history of infection, the interpretation of these findings is limited by substantial heterogeneity in study features, including the exposure, exposure dose/duration, the probiotic strain, the statistics measured, the infectious disease outcome, and the age and comorbidities of the study population. There is also substantial heterogeneity in how disease incidence was measured, with some studies relying solely on symptom report from a questionnaire. The lack of standardized RTI diagnosis, especially in older adults [85], further complicates the interpretation of these studies. The probiotics evaluated in the included studies comprised a wide variety of species and strains, both naturally occurring and experimental. It is possible that probiotics’ immune-modulating effect is strain-specific and, thus, the positive or negative findings may be related to strain-specific variation. Due to this heterogeneity, quantitative synthesis was not considered as we did not have sufficient studies with similar population types and exposures, although our qualitative synthesis was consistent with broader meta-analyses that have been attempted in the various areas [14–16, 86]. The evidence base is also limited by a lack of adjustment for the numerous factors that modulate the risk of infection, including nutrition, sleep, exercise and vaccination status. Other factors that may influence the efficacy of probiotics include genetic factors or the individual composition of gut microbiota.

Another major limitation of our review is the search terms were designed to capture the existing literature on a broad topic (i.e., dairy products and immune function) and, therefore, may have lacked the detail required to identify the universe of studies on each of the identified outcomes. The exclusion of the search term “probiotic”, for example, may have limited our search. Nevertheless, our conclusions are similar to reviews that have restricted their exposure of interest to probiotics in general [14, 16] and probiotic fermented foods specifically [15, 86]. Furthermore, our search strategy was not designed to capture the universe of studies measuring exposures assessed through food frequency questionnaires and we may have only captured those with keywords available in the abstract.

By summarizing the existing literature on this topic and providing a critical qualitative appraisal, this review plays an important role in that it provides a roadmap for valuable future research. A consortium of multicenter, randomized, placebo-controlled trials may be beneficial, with a range of specified exposure durations/doses, focused probiotic strains/dairy proteins, and clinically relevant outcomes (i.e., disease incidence based on objective antibody titers, when available) that are investigated along with longitudinal leukocyte and cytokine levels. Studies should incorporate sufficient numbers of patients to power their studies appropriately, given the background rate of infectious disease incidence in the underlying populations. Additional trials on the impact of traditional yogurt and milk would also be helpful to understand whether these products can be impactful without probiotic supplementation.

## Conclusions

This SLR identified a wide variety of potential applications for dairy products/components to improve infectious disease outcomes, with the strongest evidence available for a bioactive role for probiotics. The evidence base is diverse, with limited studies available on specific exposures and outcomes.

Probiotics delivered through dairy products represent a promising nutritional intervention for reducing the incidence and burden of CIDs (including reducing disease severity/symptoms/duration), although additional research is required. Adjuvant fermented dairy products could be an alternative program for preventing infection that is easy, acceptable and very impactful, given the substantial morbidity and economic burden associated with CIDs. Numerous potential antimicrobial applications of lactoferrin and probiotics were identified, including reducing the risk of sepsis, improving the symptomatic burden of HIV, reducing HCV burden, and improving the course of *H. pylori*, although the evidence base was small and the relevance of this research to dairy products is unknown. Coordinated research programs are recommended in each disease area where the chosen exposures and the dosing schedule are based on mechanistic research, outcomes are based on clinical measures and biomarkers are tracked longitudinally to potentially correlate with clinical outcomes.

### Supplementary Information

**Additional file 1.** PRISMA checklist.

**Additional file 2.** Leukocyte and cytokine response summary. Descriptions of the studies on leukocyte and cytokine response.

**Additional file 3:** **Supplemental Table 1.** Literature Search Strategy. **Supplemental Table 2.** ROB Assessment: Study Scoring and Determination of Quality. **Supplemental Table 3.** Dairy Products and Their Components and Leukocytes (*N*=76)^. **Supplemental Table 4.** Dairy Products and Their Components and Cytokines (*N*=47).

## Data Availability

Not applicable.

## Abbreviations

AOM: Acute otitis media
CI: Confidence interval
CID: Common infectious diseases
CRP: C-reactive protein
HCV: Hepatitis C virus
HR: Hazard ratio
ICAM: Intercellular adhesion molecule
IFN: Interferons
IL: Interleukin
IRR: Incidence rate ratio
LAB: Lactic acid bacteria
LRTI: Lower respiratory tract infection
MCP: Monocyte chemoattract protein
MFGM: Milk fat globular membrane
NA: Non-applicable
NK: Natural killer
NSS: Not statistically significant
OR: Odds ratio
PICOS: Population, intervention, comparator, outcomes, and study design
PRISMA: Preferred Reporting Items for Systematic Reviews and Meta-analyses
PROSPERO: Prospective Register of Systematic Reviews
RoB: Risk of bias
RR: Relative risk
RTI: Respiratory tract infection
SAA: Serum amyloid A
SES: Socioeconomic status
SLR: Systematic literature review
SS: Statistically significant
TGF: Transforming growth factor
TNF: Tumor necrosis factor
URTI: Upper respiratory tract infection
USDA: United States Department of Agriculture
VCAM: Vascular cellular adhesion molecule
WHOQOL: World Health Organization Quality of Life
