# Supplementary material for: The effect of bovine dairy products and their components on the incidence and natural history of infection: a systematic literature review
Source: Nutr J. 2024 Feb 27;23:26. doi: 10.1186/s12937-024-00923-7 (PMC10898086; doi:10.1186/s12937-024-00923-7)
Supplement: Supplementary file 2 — Additional file 2. Leukocyte and cytokine response summary. Descriptions of the studies on leukocyte and cytokine response. [file 12937_2024_923_MOESM2_ESM.docx]

*Leukocytes (N=76), Supplemental Table 3*

***Traditional and probiotic yogurt***

Twelve studies were identified in diverse geographical locations that evaluated the effect of traditional yogurt [1-11] or probiotic yogurt [3, 6, 8, 11, 12] on white blood cells and their components. They were all clinical trials with sample sizes <200, except one trial of 960 healthcare works in Japan [4]. The consumption of traditional or probiotic yogurt at the dosages (80-375 g daily) and durations (1-4 months) considered in these studies was not associated with a consistent enhanced immune cell response in studies of healthy adults [1-5, 8], healthy children [6, 7] or adults with pre-existing health conditions [9, 11-13]. Five studies observed increases in NK cell activity [5, 6], neutrophil levels [8], phagocytic activity [8], T-cell levels [6, 7], eosinophil levels [11] in relation to yogurt consumption, but there was no clear pattern of diet-related factors (e.g., dose or frequency, study quality) that differentiated these studies.

Healthy persons

Makino et al. (2010) investigated daily yogurt consumption (90 g) at an 8-week duration in 2005 and 12-week duration in 2007 in a population of healthy, elderly persons in Japan [5]. No difference in natural killer (NK) cell activity was found between levels at baseline and after 8 weeks; a statistically significant increase in NK cell activity, however, was observed between levels at baseline and after 12 weeks (p<0.001) [5]. Kinoshita et al. (2019) investigated the same yogurt intervention as Makino et al. in a greater quantity and for a longer duration. This study randomized healthy Japanese healthcare workers to 112 mL yogurt consumption for 16 weeks (N=479) or no treatment (N=482); no difference was found in NK cell activity between the intervention and control groups during follow-up from 2016-2017 [4].

In a double-blind, randomized clinical trial in Greece from 2014-2016, no differences were found in total white blood cell, granulocyte or lymphocyte counts between individuals receiving 150 g yogurt daily for 8 weeks with (N=31) or without olive oil polar lipid extract (OOPLE) (N=30) and those receiving low-dose yogurt (N=31, i.e., ≤150 g yogurt every 2 weeks for 8 weeks)[1]. Among subjects receiving yogurt without OOPLE, total lymphocyte counts were significantly decreased (p=0.025) after 8 weeks of treatment compared with baseline levels. In a study of young women supplemented with 454 grams of yogurt daily or no supplement, no difference was found at baseline or after 3 months of supplementation in *ex vivo* measures of cell-mediated immune function (lymphocyte proliferation and cytotoxic T-lymphocyte mediated cytotoxicity) [2].

In a randomized, double-blind trial in Spain, adults were randomized to 200 mL probiotic yogurt [*Lactobacillus (L.) gasseri* CECT5714 and *L. coryniformis* CECT5711, N=30] or traditional yogurt (N=30) for 4 weeks. No significant differences were observed between baseline levels and those at 4 weeks in any leukocyte categories for traditional yogurt, while significant increases in the percentage of neutrophils (p<0.01) and significant decreases in the percentage of lymphocytes (p<0.05), T-lymphocytes (p<0.01), T memory cells (p<0.05), and B-cells (p<0.05) were observed for probiotic yogurt; significant increases in phagocytic activity were also observed at 4 weeks for both the traditional and probiotic treatment arms (p<0.01 for both) [8]. A cross-over trial with a treatment arm of *L. plantarum* TENSIA added to yogurt (N=43) found no significant differences in total leukocyte counts, comparing levels at the beginning of the intervention to after 3 weeks of the dairy product; the control arm of this trial with traditional yogurt also found no significant differences in total leukocyte counts, comparing levels at the end of the washout period to after 3 weeks of the dairy product [3]. In another randomized clinical trial in Spain, healthy adolescents and adolescents with anorexia nervosa (AN) were given either 375 g traditional yogurt daily or 400 mL milk daily for 10 weeks [7]. In the yogurt arm of the trial, no significant difference was found in the total white blood cell count, total lymphocyte count or any lymphocyte subset after 10 weeks of daily yogurt consumption compared with baseline levels.

Persons with allergic/atopic disease:

In a cross-over trial of 21 adults with atopic disease in the US, there were no differences in cytotoxic T-cell levels, helper T-cell levels, or NK function when participants were given 8 oz yogurt daily for one month or 8 oz milk daily for one month [10]. Similarly, no significant difference in basophil levels was observed after 2 months of 80 g probiotic yogurt daily among 18 Japanese persons with atopic disease [12]. In a clinical trial of patients with Japanese cedar pollinosis randomized to traditional yogurt (N=20) or probiotic yogurt with *Bifidobacterium (B.) longum* BB536 (N=20) for 14 weeks in 2004, a significant increase in eosinophil levels (p<0.05) was observed for patients given probiotic yogurt after 14 weeks, compared to baseline levels; no significant difference was observed in eosinophil levels in the group given traditional yogurt, nor were any differences found in neutrophil, basophil, monocyte or lymphocyte levels in either the traditional or probiotic yogurt groups [11].

In a study of Spanish children with asthma and/or hay fever randomized to either 200 mL of probiotic yogurt with *L. gasseri*/*L. coryniformis* (intervention) or 200 mL of traditional yogurt (control) for 3 months, no significant change in IgE+ granulocytes, eosinophils, basophils, IgE+ lymphocytes, cytotoxic T-cells, helper T-cells, or B-cells was observed between baseline levels and levels at 3 months, in either the intervention or control group. Among children given the probiotic yogurt in the intervention group, a significant increase at 3 months compared to baseline was reported for total T-cell levels (p<0.01), regulatory T-cell levels (p<0.01) and % NK cells (p=0.03) [6].

Persons with other health conditions:

A randomized, double-blind clinical trial was conducted in Iran in 2019 among middle-aged adults with abdominal obesity who received 150 g traditional yogurt with or without vitamin D3 for 10 weeks; no difference was observed comparing total white blood cell counts before and after the intervention. Among the intervention group (yogurt with vitamin D3), a statistically significant decrease was observed in % neutrophils (p<0.001) and % lymphocytes (p=0.004) after 10 weeks, while no similar reductions were observed in the group receiving yogurt without vitamin D3 [9]. In a clinical trial of healthy adolescents and adolescents with AN in Spain, significantly higher levels were observed for the following lymphocyte subsets among AN patients after 10 weeks of daily yogurt consumption: total T-cell levels (p<0.05), cytotoxic T-cell levels (p<0.05) and helper T-cell levels (p<0.05). A significant decrease in B-cells (p<0.05) was also observed at 10 weeks compared to baseline for AN patients [7].

***Regular milk***

Six clinical trials compared various leukocyte levels at baseline to levels after 10-12 weeks of regular milk consumption, with no consistent effects on immune markers observed [5, 7, 9, 14-17].

Healthy persons

In a randomized clinical trial of healthy adults in Spain, no difference in the levels of total leukocytes or any leukocyte subset (neutrophils, eosinophils, basophils, monocytes, lymphocytes, T-cells, B-cells or NK cell %) was found for persons randomized to consume 3 cups of milk daily (N=22) after 8 weeks compared to baseline levels, although monocyte oxidative burst capacity and NK cell tumoricidal activity were significantly increased (p=0.029 and p=0.042, respectively) [16, 17]. Healthy adolescents in the clinical trial by Nova et al. (2006) had a significant increase in leukocytes, CD3+, CD8+, and CD4+ cells after 10 weeks of consuming 400 mL milk, compared to baseline [7]. Makino et al. (2010) reported a statistically significant increase in NK cell activity among elderly Japanese persons ingesting 100 mL milk daily at a 12-week duration (p<0.05), but no difference at an 8-week duration [5]. Another randomized clinical trial in elderly Norwegian persons found no difference in monocyte or lymphocyte levels after 12 weeks of consumption of protein-enriched milk [14].

A cross-over clinical trial of healthy male endurance runners in Australia evaluated the impact of a “chocolate-flavored dairy milk recovery beverage” taken 1 hour after a 2-hour running exercise. The dairy drink resulted in a significant increase in neutrophil function (p<0.001), while consumption of water resulted in a significant decrease in neutrophil function, suggesting that dairy milk consumed in the form of recovery beverage may support immune competency during a potentially immune-compromising period [18].

Persons with health conditions

In a clinical trial of anorexic adolescents in Spain receiving 400 mL milk daily for 10 weeks, significant increases (p<0.05) were observed for leukocytes and CD8+ cells (as reflected in a significant decrease in the CD4+/CD8+ ratio), but no significant changes were observed in CD3+, CD4+ and CD19+[7]. After a 10-week intervention of 200 mL low-fat milk (with or without vitamin D3) in Iranian adults with abdominal obesity, no effect was observed on white blood cell counts for either regular milk or milk with vitamin D3. A significant decrease in % neutrophils was observed among those given milk with vitamin D3 (p<0.001), but not those given milk without vitamin D3 [9]. No difference in helper T-cell levels was found after regular milk ingestion in a clinical trial of 9 persons with symptoms of Japanese cedar pollinosis (no information on dose was provided) [15].

***Fermented milk***

Seven clinical trials evaluated the impact of milk fermented with lactic acid bacterial (LAB) strains on various leukocyte subsets [15-17, 19-23]. One study of a fermented dairy drink (i.e., verum) was also identified [24]. No consistent pattern of an increase in the white blood cell response was observed with fermented milk. The seven trials generally had small sample sizes (<100) [15-17, 19-23], did not include a control arm [19-22], were conducted among patients with various immunocompromised states [15, 19-22], and reported on a diverse group of white blood cell types. Hence, the evidence base was limited.

Healthy persons

Studies that investigated fermented milk in healthy persons included subjects under physiological stress or immunosenescent conditions, including exercise [23], aging [16, 17], shift work [24], and infection [24]. Overall, these studies indicated some ability of fermented milk to modulate immune cell parameters.

A cross-over clinical trial of 25 recreational athletes reported no difference in NK cell levels between the intervention (500 mL of milk fermented with *L. casei* DN-114 001 for one month) and control periods (500 mL of regular milk for one month) up to 2 hours after an exercise stress test administered at the end of the intervention period. However, a significant decrease in NK cell levels was observed after exercise among both the intervention and control phases (p<0.05); the depression at 2 hours was significantly smaller (p<0.05) with fermented milk [23]. A randomized clinical trial in Spain included a treatment arm with 23 middle-aged adults randomized to fermented milk with the same strain of *L. casei* (DN-114 001) at a frequency of three cups per day for two months; no difference in the levels of total leukocytes or any leukocyte subset (neutrophils, eosinophils, basophils, monocytes, lymphocytes, T-cells, B-cells or NK cell %) was found when comparing levels at 8 weeks to baseline levels [16, 17]. However, there was a significant functional increment of the oxidative burst capacity of monocytes after consumption of the fermented milk with probiotic at 8 weeks compared to baseline levels, suggesting that daily ingestion of fermented milk containing *L. casei* can improve the monocyte defense ability in healthy people [16, 17]. Healthy German shift workers (n=500) were randomized to receive a dairy drink (verum, a European yogurt smoothie) fermented with the same probiotic strain (*L. casei* DN-114 001, Actimel) at 200 g daily for 84 days compared to a control diet between 2006-2007; among shift workers that did not develop an infectious disease during the course of this study, there were no differences between total leukocyte levels, granulocyte levels and NK cell levels/cytotoxic activity in the intervention and control arms, suggesting that verum did not impair homeostasis of the immune system in healthy worker subjects [24]; however, significant increases were observed for some leukocyte subsets among workers during the course of particular infections: all common infectious diseases (CID) (total leukocytes p=0.034 and NK cells <0.001); rhinopharyngitis (total leukocytes p=0.002, neutrophils p=0.002, NK cells p=0.001); sore throat (NK cells p=0.032); and lower respiratory tract infections (LRTI) (NK cells p=0.009). This suggests that verum-containing probiotic can modulate immune parameters in subjects with respiratory infections and may regulate the immune response to pathogens of the respiratory tract.

Persons with immunological conditions

Studies that investigated the impact of fermented milk on leukocyte levels in persons with immunological conditions included subjects with allergy [15, 20], malnutrition [19], and human T-cell lymphotropic virus type 1 (HTLV-1)-associated myelopathy/tropical spastic paraparesis (HAM/ TSP) [21].

In a prospective, open evaluation of fermented milk with the bacterial strain *L. casei* Shirota (LcS) in Japan, Matsuzaki et al. evaluated 10 patients with HAM/TSP who were given fermented milk twice daily for 4 weeks; no difference in helper T-cell levels was observed at the end of the intervention, but a statistically significant increase in NK cell activity was observed (p=0.015) [21].

A clinical trial of 120 children (60 HIV+ and 60 HIV-) provided 65 mL of milk fermented with *L. casei* Shirota daily for 8 weeks. The authors found a significant elevation in cytotoxic and helper T-cell levels and activation among HIV+ children, but a significant decrease in regulatory T-cells among HIV+ and HIV-negative children, when baseline levels were compared to levels post-intervention [20].

A study of 15 patients with allergic symptoms found a significant increase in the % of Th1 cells (p<0.01) and the Th1/Th2 ratio (p<0.01) after 4 weeks of consumption of 200 mL of milk fermented with *L. gasseri* [22]. Similarly, in a clinical trial of persons with Japanese cedar pollinosis, the 10 patients randomized to 8 weeks of fermented milk (*L. plantarum* HSK201) had a significant increase (p=0.047) in the percentage of Th1 cells, but no difference in the percentage of Th2 cells [15].

A clinical trial of malnourished Indian children given *L. acidophilus* LBKV-3 fermented milk in one treatment arm reported significant increases among 7-8 year-old children for neutrophils, basophils, monocytes and lymphocytes (no p-values reported) [19].

***Cheese***

Two cross-over trials of probiotic and regular cheese were identified [3, 25]. In a cross-over trial of nursing home residents in Finland, a significant increase in the proportion of granulocytes and monocytes (as well as their phagocytic activity) was observed at the end of the run-in period with one slice of regular cheese daily for 2 weeks, at the end of the intervention period with one slice of probiotic cheese (*L. rhamnosus* HN001 and *L. acidophilus* NCFM) daily for 2 weeks, and at the end of the wash-out period with one slice of regular cheese for 4 weeks, compared to baseline levels [25]. A clinical trial of healthy adults in Estonia found no difference in total leukocyte counts before and after 50 grams of probiotic cheese (*L. plantarum* TENSIA) eaten daily for 3 weeks[3].

*Dietary patterns related to dairy*

Four studies measured the relationship between leukocyte components and dietary patterns related to dairy [26, 27] or recall of cow-milk dairy consumption [28, 29]. No association was reported between CD4 counts or CD4% and a dietary cluster pattern including fruits, vegetables and low-fat dairy in a prospective cohort of US adults with HIV enrolled between 1995-2005 [26]. In a prospective cohort of healthy adults enrolled from 1991-1994 in Sweden (N=4,999), a positive association was observed between a milk-fat eating pattern and higher WBC counts (p=0.03 and p=0.06 for women and men, respectively)[27]. In two cross-sectional studies of children, no associations were found between any leukocyte subsets and maternal intake of dairy among pediatric atopic dermatitis cases in Poland [28] or milk consumption in healthy children in Greece [29].

***Probiotics***

Twenty-eight studies were identified that examined the impact of adding probiotics to cow’s milk dairy products on leukocyte levels and function by comparing responses to products with added probiotics to responses to products with no probiotics. The following genus/species of probiotics were studied in 17 studies: *Bifidobacterium lactis/animalis* [30-32], *Lactobacillus acidophilus* [13, 33], *Lactobacillus casei* [34-41], *Lactobacillus lactis* [42], *Lactobacillus paracasei* [43, 44], *Lactobacillus johnsonii* [45], *Lactobacillus reuteri* [46]*, Lactobacillus rhamnosus* [46, 47], and combinations of probiotic strains [8, 48-53]. The probiotic was fermented in milk [33-45, 49, 53, 54]/milk powder [30, 31]; the probiotic was added to yogurt in 8 studies [8, 13, 46-48, 50, 51, 55] or to yogurt smoothies/dairy drinks in 2 studies [32, 52].

Figure 2 summarizes the changes in leukocyte levels by probiotic strain type in eight studies that included a probiotic of the genus *Bifidobacterium* [11, 30-32, 50-53]. The addition of *Bifidobacterium* to dairy products resulted in no significant differences in the levels/proliferation of total white blood cells, neutrophils/eosinophils/basophils, monocytes, lymphocytes, B-cells or T-cells [31, 32, 50-53], with only one study reporting a significant increase in T-cells (p<0.05) after 3 weeks of milk powder supplementation with *B. lactis* HN019 in a cross-over trial in 30 healthy, elderly persons [31]. In three cross-over trials of *B. lactis/animalis* added to milk powder or yogurt and given to healthy adults [30-32], statistically significant increases in NK cell levels (p<0.01 [31]) and NK cell activity (p=0.043 [30], p<0.01 at all E:T ratios [51]) and cytotoxicity (p-value<0.05 [32]) at higher dosages were observed.

Twenty-two studies evaluated the impact of adding the probiotic strain *Lactobacillus* to a dairy product on white blood cell levels [8, 13, 33, 35-37, 39-51, 53-55] (Figure 2). The studies were all clinical trials with treatment durations ranging from 2-12 weeks; 15 studies enrolled healthy adult populations [8, 35-37, 39, 41-43, 45, 46, 48, 50, 51, 53, 55], while 7 studies restricted enrollment to individuals with allergic diseases [10, 33, 43, 44, 47-49, 54] or to elderly persons [40]. Comparisons between the probiotic product and the non-probiotic product found no significant differences in total levels of white blood cells, granulocytes, monocytes, lymphocytes, T-cells and B-cells, with few statistically significant increases reported [8, 25, 36, 42]. Similar to the observation with *Bifidobacterium*, however, significant increases in NK cell levels/percentages and NK cell cytotoxicity/activity were observed in a greater proportion of the studies of *Lactobacillus* than other markers of immune function (Figure 2) [8, 36, 37, 40, 51].

Eleven studies examined the phagocytic capacity of different leukocyte components, including peripheral blood mononuclear cells (PBMCs)[31], peripheral mononuclear cells (PMNs)[30, 31, 52, 56, 57], leukocytes[45, 58], granulocytes and monocytes[8, 50], and neutrophils[39]. Seven of these studies reported an increase in phagocytic capacity when levels were compared between interventions with probiotic yogurt/milk and interventions with traditional yogurt/milk[30, 31, 39, 50, 56-59], suggesting probiotics may enhance the phagocytic process.

***Milk proteins***

Nineteen clinical trials were identified with an intervention of dairy milk protein and subsequent follow-up for leukocyte levels (Supplementary Table 3) [60-78]. These studies included 5 studies of whey protein overall [66, 68, 69, 71, 74, 76, 79], 10 studies of bovine lactoferrin specifically [60-64, 67, 73, 75, 77, 78], 2 studies of casein protein [65, 66] and 2 studies of milk proteins/peptides [70, 72]. Except for 1 trial (N=198) [64], the remaining 17 trials had sample sizes less than 100 and involved supplementation with milk proteins for a duration of 5 days-12 months. Many of the trials were conducted in specific immune-challenged populations to evaluate the impact of the intervention on improving immune status (overweight persons [77], COVID patients [78], HIV patients [68, 69, 75, 80], chronic hepatitis C patients [64], cystic fibrosis [71], and cancer patients [65]).

Figure 3 describes the results of 19 studies with an intervention of dairy milk protein and subsequent follow-up for leukocyte levels. Most comparisons between intervention and control groups (or pre- vs. post-intervention levels) showed no significant changes in leukocyte totals and subsets for both healthy and immune compromised populations. For studies evaluating immune compromised populations, significant increases were found for leukocytes (p<0.05) and monocytes (p<0.05) when comparing a high-protein diet with lactoferrin supplementation for 6 days to a control diet [77] and for lymphocytes (p=0.002) when comparing a 7-day supplementation with bovine lactoferrin to placebo in hospitalized patients with COVID [78]. Among healthy populations, significant increases in immature bands of neutrophils were observed in two clinical trials of Polish adults when comparing pre- to post-intervention levels with regimens of 5-50 mg bovine lactoferrin daily for 7-10 days [62, 63]. In another clinical trial of Polish adults, lactoferrin capsules daily for 5 days resulted in a significant increase in PBMC proliferation, compared to placebo tablets [61]. Furthermore, in a clinical trial of healthy elderly persons in Japan, significant increases in the NK cell components CD16+ (p=0.03) and CD56+ (p=0.01) and NK cell cytotoxicity (p<0.05) were observed when comparing the levels observed in persons randomized to the intervention (300 mg bovine lactoferrin daily for 3 months) to the levels observed in persons randomized to placebo [73]. In a randomized clinical trial of adults in Korea, NK activity was enhanced among the persons receiving 4.2 g fermented Maillard-reactive whey protein daily for 8 weeks compared to placebo at all E:T ratios (10:1, p<0.001, 5:1, p<0.001, 2.5:1, p=0.020, and 1.25:1, p=0.010) [74, 76].

Phagocytic capacity was increased with bovine lactoferrin treatment in two clinical trials; one study found increased phagocytic capacity in neutrophils (p<0.05) with 300mg lactoferrin daily for 3 months in elderly subjects, compared to placebo [73], while another study reported increased phagocytic activity in leukocytes (p=0.01) after daily lactoferrin treatment for 4 weeks in children with HIV, compared to pre-intervention levels [60].

*Cytokines (N=47), Supplemental Table 4*

Forty-seven studies evaluated the effect of dairy products or their components on cytokine production (Supplementary Table 4); cytokines are a broad category of small proteins triggered by infection and include interleukins (IL), interferons (IFN), transforming growth factor-beta (TGF-β), and chemokines. Overall, the results of these studies were conflicting, with most studies reporting no significant impact of dairy products (including whole dairy products, probiotic specifically, and dairy proteins) on cytokine production, as described further below. Interpretation of results remained challenging as biological or clinical relevance was not considered.

***Whole dairy products***

Twelve studies investigated cytokine production and whole dairy product consumption, including traditional yogurt [2, 4, 10], regular milk [14, 81], probiotic yogurt [12, 82, 83], dairy drinks [24, 84], or multiple whole dairy products [7, 85]. Consumption of these dairy products did not result in any significant changes in interleukin levels, including IL-2 [2, 4, 10, 14, 24], IL-4 [2, 4, 10, 84], IL-5 [4], IL-10 [4, 14, 81, 82, 84], IL-12 [4, 84], IL-13 [4] or in thymus and activation-regulated chemokine (TARC) [12, 24].

There were 3 studies, however, that reported significant changes in IL levels in specific subpopulations [7, 83, 85]. A clinical trial of female adolescents (healthy and those with AN) found a significant decrease (p<0.05) in *in vitro* production of IL-2 after 10 weeks of consumption of 400 mL semi-skimmed milk daily among healthy females (compared to baseline), but a significant increase (p<0.05) in IL-2 after 10 weeks of consumption of 375 g of traditional yogurt daily (compared to baseline) among AN patients. In this study, there were no significant differences in IL-2 levels between treatments (semi-skimmed milk vs. natural yogurt) in either the healthy or AN groups [7]. A clinical trial of obese and overweight adults consuming either a probiotic yogurt with a low-calorie diet, a regular yogurt with a low-calorie diet, or a probiotic yogurt without a low-calorie diet for 8 weeks found a significant decrease (p <0.05) in IL-4 in all groups, a significant increase (p<0.05) in IL-10 in both probiotic groups, and a decrease (p <0.05) in IL-17 in the low-calorie groups after 8 weeks compared to baseline [85]. A clinical trial of rhinopathic and healthy patients fed either 450 g of yogurt or 450 g of partially skimmed milk for 4 months found that the group fed yogurt released less IL-4 in the healthy sub-group (no p-value reported) [83].

Six studies investigated IFN-gamma levels in relation to whole dairy product consumption, with conflicting findings. Three studies did not find whole dairy products (including yogurt and partially skimmed milk) resulted in a significant difference in IFN-gamma levels in healthy persons [14, 83] or persons with atopic disease [10, 83], while two studies found a significant increase [4, 7] and two studies found a significant decrease [7, 85] in IFN-gamma levels. In a clinical trial of female healthcare workers, intake of traditional yogurt fermented with *L. delbrueckii ssp. bulgaricus* OLL1073R-1 daily for 16 weeks resulted in a significant increase in IFN-gamma levels (p=0.03) compared to controls receiving no yogurt [4]. A clinical trial of a 10-week intervention with yogurt containing *L. bulgaricus* and *S. thermophiles* also found a significant increase in IFN-gamma production in health adolescents (p<0.01) and anorexia nervosa patients (p<0.01) compared to those consuming semi-skimmed milk. A 10-week intake of milk or yogurt was associated with significantly decreased IFN-gamma production in AN patients at 10 weeks compared to baseline (p<0.05) [7]. A clinical trial of obese and overweight adults consuming either a probiotic yogurt with a low-calorie diet, a regular yogurt with a low-calorie diet, or a probiotic yogurt without a low-calorie diet found a significant (p<0.05) decrease in IFN-gamma in the probiotic groups, but not in the regular groups, after 8 weeks compared to baseline [85].

***Probiotics***

Twenty-five studies investigated the relationship between cytokine production and probiotics (added to yogurts or fermented in milk), including *L. casei* [34, 39, 86-88], *B. lactis* [57], *B. animalis* [89-91], *B. longum* [11], *L. acidophilus* [13], *L. paracasei* [44], *L. lactis* [92], *L. gasseri* [49, 93], or a combination of probiotic strains [8, 49, 51, 52, 85, 94-99]. Most studies (N=22) reported no significant effects of probiotic yogurts and milks on cytokine levels, including IL-2 [13, 39, 86, 88, 90], IL-4 [6, 13, 44, 85-89, 91, 95, 99], IL-5 [87, 89, 95, 98], IL-10 [6, 11, 34, 44, 86-88, 91, 94, 95, 98, 99], IL-12 [6, 8, 86, 87, 89, 95, 96, 98], IL-13 [87, 98], IL-17 [98], interferons [11, 34, 39, 44, 85, 88, 90-92, 94, 95, 97, 98], TGF [85, 87, 95] and chemokines, including TARC [49], eotaxin [87, 95], IP-10 [95], RANTES (Regulated upon Activation, Normal [T Cell](https://www.sciencedirect.com/topics/medicine-and-dentistry/t-cell) Expressed and Presumably Secreted) [87, 95], and MIP-1a [87]. Studies of whole dairy products where a significant difference in cytokine levels was found are reported below, by healthy populations and populations with an immune challenge.

Healthy persons

The results of studies evaluating the impact of dairy products with added probiotics on cytokine production in healthy populations of adults and elderly varied in the cytokine type and in the direction of the response. One clinical trial investigating several different probiotics added to milk found that consumption of *B. animalis ssp. lactis* Bb12 (Bb12) in milk daily for 3 weeks resulted in a significant reduction in IL-2 in influenza-virus stimulated PBMC, compared to healthy adults consuming a milk-based drink containing *L. rhamnosus* GG (LGG) and *Propionibacterium freudenreichii ssp. shermanii* JS (PJS) or a placebo drink with no added probiotic for 3 weeks (p<0.001) [94]. A clinical trial of adults consuming yogurt containing *L. paracasei*, *L. casei* 431, and *L. fermentium* PCC once daily for 12 weeks had a statistically significant decrease in IFN (p<0.001) compared to yogurt with no added probiotics[99]. Another clinical trial of healthy adults consuming 120 mL of yogurt containing *L. paracasei* and *B. lactis* once daily for 12 weeks found a significant increase in IL-12 at 12 weeks compared to baseline (p<0.01). In a clinical trial of healthy elderly persons, a significant increase (p=0.036) in IFN-alpha in the intervention group consuming 180 mL of milk containing *B. lactis* was observed at week 6, compared to baseline[57].

A clinical trial of obese and overweight adults consuming probiotic yogurt containing *S. thermophile*s, *L. acidophilus*, *B. lactis* Bb12 with a low-calorie diet, a regular yogurt with a low-calorie diet, or probiotic yogurt without a low-calorie diet for 8 weeks found a significant increase (p<0.05) in IL-10 at week 8 in the probiotic yogurt group with a low-calorie diet, compared to the regular yogurt group with a low-calorie diet. A significant decrease in IL-17 (p<0.05) was also reported in the probiotic yogurt group with a low-calorie diet, compared to the regular yogurt/low-calorie diet group [85].

Persons with an immunological condition

Six studies reported significant differences in cytokine levels associated with dairy products with added probiotics in individuals with atopic disease. A double-blind, placebo-controlled parallel study of adults with birch-pollen allergy consuming one of five different probiotic yogurts containing four *L. plantarum* strains and one *L. casei* strain or a placebo for 4 weeks found a significant decrease in IL-5 (p=0.024) and IL-13 (p=0.04) and a significant increase in IL-10 (p=0.04) after 4 weeks (compared to baseline) in the groups consuming the probiotic *L. plantarum* CBS125632; there were no significant differences, however, in IL-17, IL-12, or IFN-gamma levels in any of the groups[98]. In a cross-over trial of adults with atopic dermatitis, daily intake of yogurt with added probiotic (*B. animalis subsp. lactis* LKM512) for 4 weeks resulted in a significant increase in IFN-gamma compared to baseline (p<0.005); this increase also occurred in the placebo group taking yogurt without the added probiotic (p<0.05), however, and no changes in the concentration of other cytokines (IL-4, IL-5, IL-12) with consumption of the LKM512 yogurt or placebo were reported [89]. A randomized, double-blind, placebo-controlled study in adults with allergic rhinitis, based on two 4-week cross-over periods of product consumption (*L. paracasei* ST11-fermented milk vs. placebo) and separated by a wash-out period of 6–8 weeks, reported a significant decrease in IL-5 secretion after consumption of *L. paracasei* ST11 fermented milk, comparing post-treatment to pre-treatment levels (p=0.03); this difference was not reported for IL-4, IL-8, IL-10 and IFN-gamma, however. Furthermore, IL-4, IL-5, IL-8, IL-10 and IFN-gamma levels were not significantly different between the intervention and placebo groups [44].

A clinical trial of adults with allergic rhinitis reported a significant decrease in IL-5 after consumption of a probiotic milk containing LcS daily for 2 weeks compared to milk without added probiotic with no allergic challenge (p<0.01) or during early season allergen challenge (p=0.01); a similar finding was not observed with a late season allergen challenge (p=0.05) [86]. This trial also found a significant decrease in IL-6 and IFN-gamma during the early season (p<0.01; p=0.01, respectively) and late season (p<0.01; p=0.02, respectively) allergen challenges in the treatment group compared to placebo [86]. In another clinical trial of adults with seasonal allergic rhinitis, consuming a dairy drink with the added probiotic LcS daily for 4 weeks resulted in a significant increase in IFN-gamma production compared to the control group consuming the dairy drink without the added probiotic (p=0.0351) [87].

***Dairy proteins and fats***

Nine studies investigated the impact of dairy proteins and fats, including bovine lactoferrin [60, 67, 75, 100, 101], total whey protein [67, 74, 102], casein protein [102] and milk phospholipids [103] on cytokine levels. One study investigated milk phospholipids and found no impact of 3 grams of phospholipid in milk on macrophage-derived chemokine compared to normal whole milk [103]. For the following cytokines, no significant differences were observed when levels were compared to pre-intervention levels, levels observed with no dairy protein/fat treatment, or levels observed with placebo: IL-2 [67, 104], IL-4 [67], IL-16 [103], or interferons [67, 74, 102]. There were no studies investigating the effect of dairy proteins on IL-13 or IL-17.

Regarding other cytokines, results were conflicting. Only one study investigated the effect of dairy protein on IL-5 levels. This clinical trial of adults with chronic liver disease consuming whey and casein protein daily for 15 days had a statistically significant increase in IL-5 (p=0.027), the chemokine IP-10/CXCL10 (p=0.022), and eotaxin-1/CCL11 (p=0.031), compared to baseline levels [102]. Studies investigating the effect of dairy proteins on IL-10 and IL-12 were conducted in diverse populations with conflicting results. A clinical trial of post-menopausal women consuming bovine lactoferrin daily for 6 months had a significant increase in IL-10 (p=0.0235) and decrease in TGF-beta (p=0.0072), compared to placebo by the end of the study [100]. A clinical trial of HIV+/ART-naïve children consuming 1 g of bovine lactoferrin every eight hours for 4 weeks had a significant increase in the median IL-12/IL-10 ratio (p=0.001) [60]. A clinical trial of healthy adults consuming 4.2 g of fermented Maillard-reactive whey protein daily for 8 weeks had a significant increase in IL-12 from baseline to 8 weeks, compared to the change for placebo (p=0.004) [74]. No significant differences were reported, however, in 4 other studies of dairy proteins and IL-10 [67, 75, 101, 102] and one study of dairy proteins and IL-12 [104].

***Dietary patterns related to dairy***

One study evaluated the impact of dietary patterns related to dairy consumption on cytokine levels. In a case-control study of asthmatic children, a statistically significant increase (p<0.05) was found in IL-17F levels associated with the consumption of dairy products [105].

1. Antonopoulou S, Detopoulou M, Fragopoulou E, Nomikos T, Mikellidi Y, M K, et al. Consumption of yogurt enriched with polar lipids from olive oil by-products reduces platelet sensitivity against platelet activating factor and inflammatory indices: A randomized, double-blind clinical trial. Human Nutrition and Metabolism. 2022;28.

2. Campbell CG, Chew BP, Luedecke LO, Shultz TD. Yogurt consumption does not enhance immune function in healthy premenopausal women. Nutrition and Cancer. 2000;37(1):27-35.

3. Hütt P, Songisepp E, Rätsep M, Mahlapuu R, Kilk K, Mikelsaar M. Impact of probiotic Lactobacillus plantarum TENSIA in different dairy products on anthropometric and blood biochemical indices of healthy adults. Beneficial Microbes. 2015;6(3):233-43.

4. Kinoshita T, Maruyama K, Suyama K, Nishijima M, Akamatsu K, Jogamoto A, et al. The effects of OLL1073R-1 yogurt intake on influenza incidence and immunological markers among women healthcare workers: a randomized controlled trial. Food & function. 2019;10(12):8129-36.

5. Makino S, Ikegami S, Kume A, Horiuchi H, Sasaki H, Orii N. Reducing the risk of infection in the elderly by dietary intake of yoghurt fermented with Lactobacillus delbrueckii ssp. bulgaricus OLL1073R-1. British Journal of Nutrition. 2010;104(7):998-1006.

6. Martínez-Cañavate A, Sierra S, Lara-Villoslada F, Romero J, Maldonado J, Boza J, et al. A probiotic dairy product containing L. gasseri CECT5714 and L. coryniformis CECT5711 induces immunological changes in children suffering from allergy. Pediatric Allergy and Immunology. 2009;20(6):592-600.

7. Nova E, Toro O, Varela P, López-Vidriero I, Morandé G, Marcos A. Effects of a nutritional intervention with yogurt on lymphocyte subsets and cytokine production capacity in anorexia nervosa patients. European Journal of Nutrition. 2006;45(4):225-33.

8. Olivares M, Díaz-Ropero MP, Gómez N, Lara-Villoslada F, Sierra S, Maldonado JA, et al. The consumption of two new probiotic strains, Lactobacillus gasseri CECT 5714 and Lactobacillus coryniformis CECT 5711, boosts the immune system of healthy humans. International Microbiology. 2006;9(1):47-52.

9. Sharifan P, Rashidmayvan M, Khorasanchi Z, Darroudi S, Heidari A, Hoseinpoor F, et al. Efficacy of low-fat milk and yogurt fortified with vitamin D3 on systemic inflammation in adults with abdominal obesity. Journal of health, population, and nutrition. 2022;41(1):8.

10. Wheeler JG, Bogle ML, Shema SJ, Shirrell MA, Stine KC, Pittler AJ, et al. Impact of dietary yogurt on immune function. American Journal of the Medical Sciences. 1997;313(2):120-3.

11. Xiao JZ, Kondo S, Yanagisawa N, Takahashi N, Odamaki T, Iwabuchi N, et al. Effect of probiotic Bifidobacterium longum BBS36 in relieving clinical symptoms and modulating plasma cytokine levels of japanese cedar pollinosis during the pollen season. A randomized double-blind, placebo-controlled trial. Journal of Investigational Allergology and Clinical Immunology. 2006;16(2):86-93.

12. Suzuki T, Nishiyama K, Kawata K, Sugimoto K, Isome M, Suzuki S, et al. Effect of the lactococcus lactis 11/19-B1 strain on atopic dermatitis in a clinical test and mouse model. Nutrients. 2020;12(3).

13. Wheeler JG, Shema SJ, Bogle ML, Shirrell MA, Burks AW, Pittler A, et al. Immune and clinical impact of Lactobacillus acidophilus on asthma. Annals of Allergy, Asthma and Immunology. 1997;79(3):229-33.

14. Gjevestad GO, Ottestad I, Biong AS, Iversen PO, Retterstøl K, Raastad T, et al. Consumption of protein-enriched milk has minor effects on inflammation in older adults—A 12-week double-blind randomized controlled trial. Mechanisms of Ageing and Development. 2017;162:1-8.

15. Hasegawa T, Hirakawa K, Matsumoto T, Toki S, Maeyama Y, Morimatsu F. Efficacy of Lactobacillus plantarum strain HSK201 in relief from Japanese cedar pollinosis. Bioscience, Biotechnology and Biochemistry. 2009;73(12):2626-31.

16. Parra D, De Morentin BM, Cobo JM, Mateos A, Martinez JA. Monocyte function in healthy middle-aged people receiving fermented milk containing Lactobacillus casei. J Nutr Health Aging. 2004;8(4):208-11.

17. Parra MD, Martínez de Morentin BE, Cobo JM, Mateos A, Martínez JA. Daily ingestion of fermented milk containing Lactobacillus casei DN114001 improves innate-defense capacity in healthy middle-aged people. J Physiol Biochem. 2004;60(2):85-91.

18. Costa RJS, Camões-Costa V, Snipe RMJ, Dixon D, Russo I, Huschtscha Z. The impact of a dairy milk recovery beverage on bacterially stimulated neutrophil function and gastrointestinal tolerance in response to hypohydration inducing exercise stress. International Journal of Sport Nutrition and Exercise Metabolism. 2020;30(4):237-48.

19. Hajare ST. Effects of potential probiotic strains LBKV-3 on Immune Cells responses in Malnutrite children: a double-blind, randomized,Controlled trial. Journal of Immunoassay and Immunochemistry. 2021;42(5):453-66.

20. Ishizaki A, Bi X, Van Nguyen L, Matsuda K, Pham HV, Phan CTT, et al. Effects of short-term probiotic ingestion on immune profiles and microbial translocation among HIV-1-infected Vietnamese children. International Journal of Molecular Sciences. 2017;18(10).

21. Matsuzaki T, Saito M, Usuku K, Nose H, Izumo S, Arimura K, et al. A prospective uncontrolled trial of fermented milk drink containing viable Lactobacillus casei strain Shirota in the treatment of HTLV-1 associated myelopathy/tropical spastic paraparesis. Journal of the Neurological Sciences. 2005;237(1-2):75-81.

22. Morita H, He F, Kawase M, Kubota A, Hiramatsu M, Kurisaki JI, et al. Preliminary human study for possible alteration of serum immunoglobulin E production in perennial allergic rhinitis with fermented milk prepared with Lactobacillus gasseri TMC0356. Microbiology and Immunology. 2006;50(9):701-6.

23. Pujol P, Huguet J, Drobnic F, Banquells M, Ruiz O, Galilea P, et al. The effect of fermented milk containing Lactobacillus casei on the immune response to exercise. Sports Medicine, Training and Rehabilitation. 2000;9(3):209-23.

24. Guillemard E, Tanguy J, Flavigny A, e la Motte S, Schrezenmeir J. Effects of consumption of a fermented dairy product containing the probiotic Lactobacillus casei DN-114 001 on common respiratory and gastrointestinal infections in shift workers in a randomized controlled trial. Journal of the American College of Nutrition. 2010;29(5):455-68.

25. Ibrahim F, Ruvio S, Granlund L, Salminen S, Viitanen M, Ouwehand AC. Probiotics and immunosenescence: Cheese as a carrier. FEMS Immunology and Medical Microbiology. 2010;59(1):53-9.

26. Hendricks KM, Mwamburi DM, Newby PK, Wanke CA. Dietary patterns and health and nutrition outcomes in men living with HIV infection. American Journal of Clinical Nutrition. 2008;88(6):1584-92.

27. Hlebowicz J, Persson M, Gullberg B, Sonestedt E, Wallström P, Drake I, et al. Food patterns, inflammation markers and incidence of cardiovascular disease: The Malmö Diet and Cancer study. Journal of Internal Medicine. 2011;270(4):365-76.

28. Milewska-Wróbel D, Lis-Święty A. Does maternal diet during pregnancy influence clinical and laboratory characteristics of infantile-onset atopic dermatitis? Eur Ann Allergy Clin Immunol. 2020;52(6):277-9.

29. Moschonis G, Van den Heuvel EGHM, Mavrogianni C, Singh-Povel CM, Leotsinidis M, Manios Y. Associations of milk consumption and vitamin B2 and b12 derived from milk with fitness, anthropometric and biochemical indices in children. The healthy growth study. Nutrients. 2016;8(10).

30. Chiang BL, Sheih YH, Wang LH, Liao CK, Gill HS. Enhancing immunity by dietary consumption of a probiotic lactic acid bacterium (Bifidobacterium lactis HN019): Optimization and definition of cellular immune responses. European Journal of Clinical Nutrition. 2000;54(11):849-55.

31. Gill HS, Rutherfurd KJ, Cross ML, Gopal PK. Enhancement of immunity in the elderly by dietary supplementation with the probiotic Bifidobactedum lactis HN019. American Journal of Clinical Nutrition. 2001;74(6):833-9.

32. Meng H, Ba Z, Lee Y, Peng J, Lin J, Fleming JA, et al. Consumption of Bifidobacterium animalis subsp. lactis BB-12 in yogurt reduced expression of TLR-2 on peripheral blood-derived monocytes and pro-inflammatory cytokine secretion in young adults. European Journal of Nutrition. 2017;56(2):649-61.

33. Ishida Y, Nakamura F, Kanzato H, Sawada D, Hirata H, Nishimura A, et al. Clinical effects of Lactobacillus acidophilus strain L-92 on perennial allergic rhinitis: a double-blind, placebo-controlled study. Journal of dairy science. 2005;88(2):527-33.

34. Elmadfa I, Klein P, Meyer AL. Immune-stimulating effects of lactic acid bacteria in vivo and in vitro. Proc Nutr Soc. 2010;69(3):416-20.

35. Kato-Kataoka A, Nishida K, Takada M, Kawai M, Kikuchi-Hayakawa H, Suda K, et al. Fermented Milk Containing Lactobacillus casei Strain Shirota Preserves the Diversity of the Gut Microbiota and Relieves Abdominal Dysfunction in Healthy Medical Students Exposed to Academic Stress. Applied and environmental microbiology. 2016;82(12):3649-58.

36. Marcos A, Wärnberg J, Nova E, Gómez S, Alvarez A, Alvarez R, et al. The effect of milk fermented by yogurt cultures plus Lactobacillus casei DN-114001 on the immune response of subjects under academic examination stress. European Journal of Nutrition. 2004;43(6):381-9.

37. Morimoto K, Takeshita T, Nanno M, Tokudome S, Nakayama K. Modulation of natural killer cell activity by supplementation of fermented milk containing Lactobacillus casei in habitual smokers. Preventive Medicine. 2005;40(5):589-94.

38. Shida K, Sato T, Iizuka R, Hoshi R, Watanabe O, Igarashi T, et al. Daily intake of fermented milk with Lactobacillus casei strain Shirota reduces the incidence and duration of upper respiratory tract infections in healthy middle-aged office workers. European Journal of Nutrition. 2017;56(1):45-53.

39. Spanhaak S, Havenaar R, Schaafsma G. The effect of consumption of milk fermented by Lactobacillus casei strain Shirota on the intestinal microflora and immune parameters in humans. European Journal of Clinical Nutrition. 1998;52(12):899-907.

40. Takeda K, Suzuki T, Shimada SI, Shida K, Nanno M, Okumura K. Interleukin-12 is involved in the enhancement of human natural killer cell activity by Lactobacillus casei Shirota. Clinical and Experimental Immunology. 2006;146(1):109-15.

41. Tiollier E, Chennaoui M, Gomez-Merino D, Drogou C, Filaire E, Guezennec CY. Effect of a probiotics supplementation on respiratory infections and immune and hormonal parameters during intense military training. Military Medicine. 2007;172(9):1006-11.

42. Kimoto-Nira H, Nagakura Y, Kodama C, Shimizu T, Okuta M, Sasaki K, et al. Effects of ingesting milk fermented by Lactococcus lactis H61 on skin health in young women: a randomized double-blind study. Journal of dairy science. 2014;97(9):5898-903.

43. Yoon JY, Cha JM, Hong SS, Kim HK, Kwak MS, Jeon JW, et al. Fermented milk containing Lactobacillus paracasei and Glycyrrhiza glabra has a beneficial effect in patients with Helicobacter pylori infection: A randomized, double-blind, placebo-controlled study. Medicine. 2019;98(35):e16601.

44. Wassenberg J, Nutten S, Audran R, Barbier N, Aubert V, Moulin J, et al. Effect of Lactobacillus paracasei ST11 on a nasal provocation test with grass pollen in allergic rhinitis. Clinical and Experimental Allergy. 2011;41(4):565-73.

45. Fukushima Y, Miyaguchi S, Yamano T, Kaburagi T, Iino H, Ushida K, et al. Improvement of nutritional status and incidence of infection in hospitalised, enterally fed elderly by feeding of fermented milk containing probiotic Lactobacillus johnsonii La1 (NCC533). British Journal of Nutrition. 2007;98(5):969-77.

46. Jones ML, Martoni CJ, Tamber S, Parent M, Prakash S. Evaluation of safety and tolerance of microencapsulated Lactobacillus reuteri NCIMB 30242 in a yogurt formulation: A randomized, placebo-controlled, double-blind study. Food and Chemical Toxicology. 2012;50(6):2216-23.

47. Hummelen R, Hemsworth J, Changalucha J, Butamanya NL, Hekmat S, Habbema JD, et al. Effect of micronutrient and probiotic fortified yogurt on immune-function of anti-retroviral therapy naive HIV patients. Nutrients. 2011;3(10):897-909.

48. Anukam KC, Osazuwa EO, Osadolor HB, Bruce AW, Reid G. Yogurt containing probiotic Lactobacillus rhamnosus GR-1 and L. reuteri RC-14 helps resolve moderate diarrhea and increases CD4 count in HIV/AIDS patients. Journal of Clinical Gastroenterology. 2008;42(3):239-43.

49. Kawase M, He F, Kubota A, Hiramatsu M, Saito H, Ishii T, et al. Effect of fermented milk prepared with two probiotic strains on Japanese cedar pollinosis in a double-blind placebo-controlled clinical study. International Journal of Food Microbiology. 2009;128(3):429-34.

50. Klein A, Friedrich U, Vogelsang H, Jahreis G. Lactobacillus acidophilus 74-2 and Bifidobacterium animalis subsp lactis DGCC 420 modulate unspecific cellular immune response in healthy adults. European Journal of Clinical Nutrition. 2008;62(5):584-93.

51. Lee A, Lee YJ, Yoo HJ, Kim M, Chang Y, Lee DS, et al. Consumption of dairy yogurt containing Lactobacillus paracasei ssp. paracasei, Bifidobacterium animalis ssp. lactis and Heat-Treated Lactobacillus plantarum improves immune function including natural killer cell activity. Nutrients. 2017;9(6).

52. Rizzardini G, Eskesen D, Calder PC, Capetti A, Jespersen L, Clerici M. Evaluation of the immune benefits of two probiotic strains Bifidobacterium animalis ssp. lactis, BB-12® and Lactobacillus paracasei ssp. paracasei, L. casei 431® in an influenza vaccination model: A randomised, double-blind, placebo-controlled study. British Journal of Nutrition. 2012;107(6):876-84.

53. Schiffrin EJ, Brassart D, Servin AL, Rochat F, Donnet-Hughes A. Immune modulation of blood leukocytes in humans by lactic acid bacteria: criteria for strain selection. Am J Clin Nutr. 1997;66(2):515s-20s.

54. Ishida Y, Nakamura F, Kanzato H, Sawada D, Yamamoto N, Kagata H, et al. Effect of milk fermented with Lactobacillus acidophilus strain L-92 on symptoms of japanese cedar pollen allergy: A randomized placebo-controlled trial. Bioscience, Biotechnology and Biochemistry. 2005;69(9):1652-60.

55. Meyer AL, Micksche M, Herbacek I, Elmadfa I. Daily intake of probiotic as well as conventional yogurt has a stimulating effect on cellular immunity in young healthy women. Annals of Nutrition and Metabolism. 2006;50(3):282-9.

56. Sheih YH, Chiang BL, Wang LH, Liao CK, Gill HS. Systemic immunity-enhancing effects in healthy subjects following dietary consumption of the lactic acid bacterium Lactobacillus rhamnosus HN001. Journal of the American College of Nutrition. 2001;20(2):149-56.

57. Arunachalam K, Gill H, Chandra RK. Enhancement of natural immune function by dietary consumption of Bifidobacterium lactis (HN019). European Journal of Clinical Nutrition. 2000;54(3):263-7.

58. Donnet-Hughes A, Rochat F, Serrant P, Aeschlimann JM, Schiffrin EJ. Modulation of nonspecific mechanisms of defense by lactic acid bacteria: effective dose. Journal of dairy science. 1999;82(5):863-9.

59. Schiffrin EJ, Parlesak A, Bode C, Bode JC, van't Hof MA, Grathwohl D, et al. Probiotic yogurt in the elderly with intestinal bacterial overgrowth: Endotoxaemia and innate immune functions. British Journal of Nutrition. 2009;101(7):961-6.

60. Zuccotti GV, Vigano A, Borelli M, Saresella M, Giacomet V, Clerici M. Modulation of innate and adaptive immunity by lactoferrin in human immunodeficiency virus (HIV)-infected, antiretroviral therapy-naïve children. International Journal of Antimicrobial Agents. 2007;29(3):353-5.

61. Zimecki M, Właszczyk A, Wojciechowski R, Dawiskiba J, Kruzel M. Lactoferrin regulates the immune responses in post-surgical patients. Archivum Immunologiae et Therapiae Experimentalis. 2001;49(4):325-33.

62. Zimecki M, Właszczyk A, Cheneau P, Brunel AS, Mazurier J, Spik G, et al. Immunoregulatory effects of a nutritional preparation containing bovine lactoferrin taken orally by healthy individuals. Archivum Immunologiae et Therapiae Experimentalis. 1998;46(4):231-40.

63. Zimecki M, Spiegel K, Właszczyk A, Kübler A, Kruzel ML. Lactoferrin increases the output of neutrophil precursors and attenuates the spontaneous production of TNF-α and IL-6 by peripheral blood cells. Archivum Immunologiae et Therapiae Experimentalis. 1999;47(2):113-8.

64. Ueno H, Sato T, Yamamoto S, Tanaka K, Ohkawa S, Takagi H, et al. Randomized, double-blind, placebo-controlled trial of bovine lactoferrin in patients with chronic hepatitis C. Cancer Sci. 2006;97(10):1105-10.

65. Rohr UD, Li WW, Ziqiang H, Wainright W, Schindler AE. The effect of fermented soy (FSWW08) on blood hematology and cachexia in cancer patients. Hormone Molecular Biology and Clinical Investigation. 2012;12(3):407-18.

66. Rankin JW, Shute M, Heffron SP, Saker KE. Energy restriction but not protein source affects antioxidant capacity in athletes. Free Radical Biology and Medicine. 2006;41(6):1001-9.

67. Mulder AM, Connellan PA, Oliver CJ, Morris CA, Stevenson LM. Bovine lactoferrin supplementation supports immune and antioxidant status in healthy human males. Nutrition Research. 2008;28(9):583-9.

68. Moreno YF, Sgarbieri VC, Silva MN, Toro AA, Vilela MM. Features of whey protein concentrate supplementation in children with rapidly progressive HIV infection. J Trop Pediatr. 2006;52(1):34-8.

69. Micke P, Beeh KM, Buhl R. Effects of long-term supplementation with whey proteins on plasma glutathione levels of HIV-infected patients. European Journal of Nutrition. 2002;41(1):12-8.

70. Lee YM, Skurk T, Hennig M, Hauner H. Effect of a milk drink supplemented with whey peptides on blood pressure in patients with mild hypertension. European Journal of Nutrition. 2007;46(1):21-7.

71. Lands LC, Iskandar M, Beaudoin N, Meehan B, Dauletbaev N, Berthiuame Y. Dietary supplementation with pressurized whey in patients with cystic fibrosis. Journal of Medicinal Food. 2010;13(1):77-82.

72. Kreider RB, Iosia M, Cooke M, Hudson G, Rasmussen C, Chen H, et al. Bioactive properties and clinical safety of a novel milk protein peptide. Nutrition Journal. 2011;10(1).

73. Kawakami H, Park H, Park S, Kuwata H, Shephard RJ, Aoyagi Y. Effects of enteric-coated lactoferrin supplementation on the immune function of elderly individuals: a randomised, double-blind, placebo-controlled trial. International Dairy Journal. 2015;47:79-85.

74. Kang M, Oh NS, Kim M, Ahn HY, Yoo HJ, Sun M, et al. Supplementation of fermented Maillard-reactive whey protein enhances immunity by increasing NK cell activity. Food & function. 2017;8(4):1718-25.

75. Ishii K, Takamura N, Shinohara M, Wakui N, Shin H, Sumino Y, et al. Long-term follow-up of chronic hepatitis C patients treated with oral lactoferrin for 12 months. Hepatol Res. 2003;25(3):226-33.

76. Han Y, Lee S, Lee JH, Yoo HJ. Potential Mechanisms of Improved Activity of Natural Killer Cells Induced by the Consumption of F-MRP for 8 weeks. Molecular nutrition & food research. 2021;65(13):e2100337.

77. Feidantsis K, Methenitis S, Ketselidi K, Vagianou K, Skepastianos P, Hatzitolios A, et al. Comparison of short-term hypocaloric high-protein diets with a hypocaloric Mediterranean diet: Effect on body composition and health-related blood markers in overweight and sedentary young participants. Nutrition. 2021;91-92.

78. Algahtani FD, Elabbasy MT, Samak MA, Adeboye AA, Yusuf RA, Ghoniem ME. The Prospect of Lactoferrin Use as Adjunctive Agent in Management of SARS-CoV-2 Patients: A Randomized Pilot Study. Medicina (Kaunas). 2021;57(8).

79. Hulmi JJ, Myllymäki T, Tenhumäki M, Mutanen N, Puurtinen R, Paulsen G, et al. Effects of resistance exercise and protein ingestion on blood leukocytes and platelets in young and older men. European Journal of Applied Physiology. 2010;109(2):343-53.

80. Zuccotti GV, Salvini F, Riva E, Agostoni C. Oral lactoferrin in HIV-1 vertically infected children: An observational follow-up of plasma viral load and immune parameters. Journal of International Medical Research. 2006;34(1):88-94.

81. Russo I, Della Gatta PA, Garnham A, Porter J, Burke LM, Costa RJS. The Effects of an Acute "Train-Low" Nutritional Protocol on Markers of Recovery Optimization in Endurance-Trained Male Athletes. International journal of sports physiology and performance. 2021;16(12):1764-76.

82. Yang YJ, Sheu BS. Probiotics-Containing Yogurts Suppress Helicobacter pylori Load and Modify Immune Response and Intestinal Microbiota in the Helicobacter pylori-Infected Children. Helicobacter. 2012;17(4):297-304.

83. Aldinucci C, Bellussi L, Monciatti G, Passàli GC, Salerni L, Passàli D, et al. Effects of dietary yoghurt on immunological and clinical parameters of rhinopathic patients. European Journal of Clinical Nutrition. 2002;56(12):1155-61.

84. Falasca K, Vecchiet J, Ucciferri C, Di Nicola M, D'Angelo C, Reale M. Effect of Probiotic Supplement on Cytokine Levels in HIV-Infected Individuals: A Preliminary Study. Nutrients. 2015;7(10):8335-47.

85. Zarrati M, Shidfar F, Nourijelyani K, Mofid V, Hossein zadeh-Attar MJ, Bidad K, et al. Lactobacillus acidophilus La5, Bifidobacterium BB12, and Lactobacillus casei DN001 modulate gene expression of subset specific transcription factors and cytokines in peripheral blood mononuclear cells of obese and overweight people. BioFactors. 2013;39(6):633-43.

86. Ivory K, Chambers SJ, Pin C, Prieto E, Arqués JL, Nicoletti C. Oral delivery of Lactobacillus casei Shirota modifies allergen-induced immune responses in allergic rhinitis. Clinical and Experimental Allergy. 2008;38(8):1282-9.

87. Ivory K, Wilson AM, Sankaran P, Westwood M, McCarville J, Brockwell C, et al. Oral delivery of a probiotic induced changes at the nasal mucosa of seasonal allergic rhinitis subjects after local allergen challenge: A randomised clinical trial. PLoS ONE. 2013;8(11).

88. Meyer AL, Elmadfa I, Herbacek I, Micksche M. Probiotic, as well as conventional yogurt, can enhance the stimulated production of proinflammatory cytokines. Journal of Human Nutrition and Dietetics. 2007;20(6):590-8.

89. Matsumoto M, Aranami A, Ishige A, Watanabe K, Benno Y. LKM512 yogurt consumption improves the intestinal environment and induces the T-helper type 1 cytokine in adult patients with intractable atopic dermatitis. Clinical and Experimental Allergy. 2007;37(3):358-70.

90. Meng H, Lee Y, Ba Z, Peng J, Lin J, Boyer AS, et al. Consumption of Bifidobacterium animalis subsp. lactis BB-12 impacts upper respiratory tract infection and the function of NK and T cells in healthy adults. Molecular nutrition & food research. 2016;60(5):1161-71.

91. Zhang H, Miao J, Su M, Liu BY, Liu Z. Effect of fermented milk on upper respiratory tract infection in adults who lived in the haze area of Northern China: a randomized clinical trial. Pharmaceutical Biology. 2021;59(1):647-52.

92. Sugimura T, Takahashi H, Jounai K, Ohshio K, Kanayama M, Tazumi K, et al. Effects of oral intake of plasmacytoid dendritic cells-stimulative lactic acid bacterial strain on pathogenesis of influenza-like illness and immunological response to influenza virus. British Journal of Nutrition. 2015;114(5):727-33.

93. Martínez-Cañavate A, Sierra S, Lara-Villoslada F, Romero J, Maldonado J, Boza J, et al. A probiotic dairy product containing L. gasseri CECT5714 and L. coryniformis CECT5711 induces immunological changes in children suffering from allergy. Pediatr Allergy Immunol. 2009;20(6):592-600.

94. Kekkonen RA, Lummela N, Karjalainen H, Latvala S, Tynkkynen S, Järvenpää S, et al. Probiotic intervention has strain-specific anti-inflammatory effects in healthy adults. World Journal of Gastroenterology. 2008;14(13):2029-36.

95. Koyama T, Kirjavainen PV, Fisher C, Anukam K, Summers K, Hekmat S, et al. Development and pilot evaluation of a novel probiotic mixture for the management of seasonal allergic rhinitis. Canadian Journal of Microbiology. 2010;56(9):730-8.

96. Lorea Baroja M, Kirjavainen PV, Hekmat S, Reid G. Anti-inflammatory effects of probiotic yogurt in inflammatory bowel disease patients. Clinical and Experimental Immunology. 2007;149(3):470-9.

97. Nishiyama K, Kobayashi T, Sato Y, Watanabe Y, Kikuchi R, Kanno R, et al. A double-blind controlled study to evaluate the effects of yogurt enriched with lactococcus lactis 11/19-b1 and bifidobacterium lactis on serum low-density lipoprotein level and antigen-specific interferon-γ releasing ability. Nutrients. 2018;10(11).

98. Snel J, Vissers YM, Smit BA, Jongen JM, van der Meulen ET, Zwijsen R, et al. Strain-specific immunomodulatory effects of Lactobacillus plantarum strains on birch-pollen-allergic subjects out of season. Clin Exp Allergy. 2011;41(2):232-42.

99. Zhang H, Yeh C, Jin Z, Ding L, Liu BY, Zhang L, et al. Prospective study of probiotic supplementation results in immune stimulation and improvement of upper respiratory infection rate. Synthetic and Systems Biotechnology. 2018;3(2):113-20.

100. Bharadwaj S, Naidu TAG, Betageri GV, Prasadarao NV, Naidu AS. Inflammatory responses improve with milk ribonuclease-enriched lactoferrin supplementation in postmenopausal women. Inflammation Research. 2010;59(11):971-8.

101. Campione E, Lanna C, Cosio T, Rosa L, Conte MP, Iacovelli F, et al. Lactoferrin as antiviral treatment in COVID-19 management: Preliminary evidence. International Journal of Environmental Research and Public Health. 2021;18(20).

102. Mizubuti YGG, Vieira ELM, Silva TA, D'Alessandro MO, Generoso SV, Teixeira AL, et al. Comparing the effects of whey and casein supplementation on nutritional status and immune parameters in patients with chronic liver disease: A randomised double-blind controlled trial. British Journal of Nutrition. 2021;125(7):768-79.

103. Keller S, Le HY, Rödiger C, Hipler UC, Kertscher R, Malarski A, et al. Supplementation of a dairy drink enriched with milk phospholipids in patients with atopic dermatitis - A double-blind, placebo-controlled, randomized, cross-over study. Clinical Nutrition. 2014;33(6):1010-6.

104. Micke P, Beeh KM, Schlaak JF, Buhl R. Oral supplementation with whey proteins increases plasma glutathione levels of HIV-infected patients. European Journal of Clinical Investigation. 2001;31(2):171-8.

105. Han YY, Forno E, Brehm JM, Acosta-Pérez E, Alvarez M, Colón-Semidey A, et al. Diet, interleukin-17, and childhood asthma in Puerto Ricans. Ann Allergy Asthma Immunol. 2015;115(4):288-93.e1.
